# Oxidative Carboxylation of Lignin: Exploring Reactivity of Different Lignin Types

**DOI:** 10.1021/acs.biomac.4c00326

**Published:** 2024-06-13

**Authors:** Fika Andriani, Martin Lawoko

**Affiliations:** † Division of Wood Chemistry and Pulp Technology, Department of Fiber and Polymer Technology, School of Chemistry, Biotechnology and Health, KTH Royal Institute of Technology, SE-100 44 Stockholm, Sweden; ‡ Wallenberg Wood Science Center, Department of Fiber and Polymer Technology, School of Chemistry, Biotechnology and Health, KTH Royal Institute of Technology, SE-100 44 Stockholm, Sweden

## Abstract

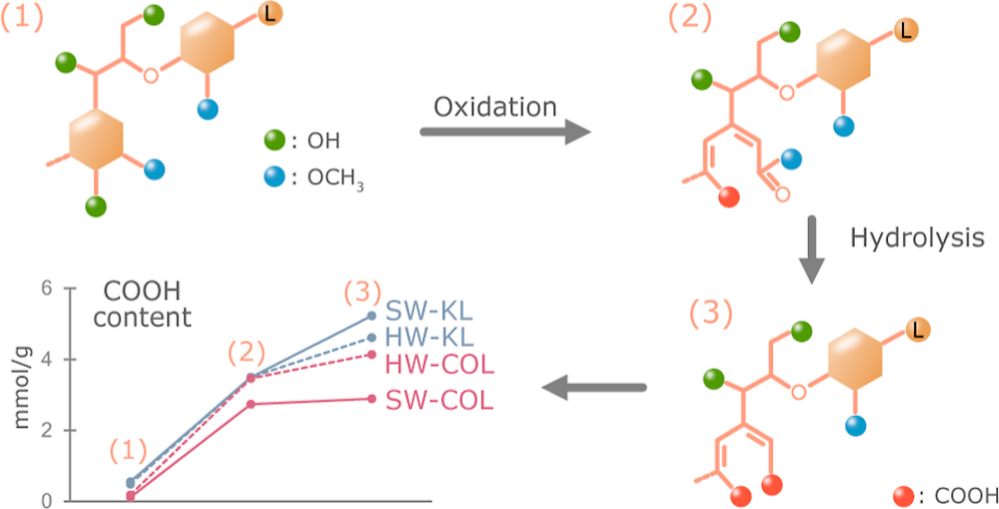

The increased interest in the utilization of lignin in biobased applications is evident from the rise in lignin valorization studies. The present study explores the responsiveness of lignin toward oxidative valorization using acetic acid and hydrogen peroxide. The pristine lignins and their oxidized equivalents were analyzed comprehensively using NMR and SEC. The study revealed ring opening of phenolic rings yielding muconic acid- and ester-end groups and side-chain oxidations of the benzylic hydroxyls. Syringyl units were more responsive to these reactions than guaiacyl units. The high selectivity of the reaction yielded oligomeric oxidation products with a narrower dispersity than pristine lignins. Mild alkaline hydrolysis of methyl esters enhanced the carboxylic acid content of oxidized lignin, presenting the potential to adjust the carboxylic acid content of lignin. While oxidation reactions in lignin valorization are well documented, this study showed the feasibility of employing optimized oxidation conditions to engineer tailored lignin-based material precursors.

## Introduction

Addressing the critical environmental challenges and moving toward sustainable methods is key. Turning to renewable resources, like biomass feedstocks, is a critical step in reducing our dependency on finite fossil resources.^1,2^ Lignin, a biomacromolecule found in plant biomass, is characterized by its abundant phenolic compounds,^3^ high thermal stability, and excellent mechanical properties and is primed to play a significant role in the development of advanced materials.^4−11^

Representing 15–30% of plant biomass by weight, lignin’s content varies significantly across different species.^12,13^ Lignin is a heterogenic natural polymer consisting of three aromatic monolignols: *p*-coumaryl, coniferyl, and sinapyl alcohols, which are precursors to the *p*-hydroxyphenyl (H), guaiacyl (G), and syringyl (S) units, respectively, which together constitute the complex structure of the lignin molecule.^14−16^ The unique composition of these units is directly influenced by the plant origin, leading to notable differences between hardwood and softwood lignins in both structure and properties.

Nevertheless, the structure and properties of lignin are also affected by the extraction method to obtain lignin. Among the numerous methods, the kraft process stands out as a time-tested approach for delignification to produce a chemical pulp. This process yields kraft lignin as a byproduct with a complex structure due to significant alterations to the native lignin.^17−19^ Consequently, the detailed structure of this technical lignin remains poorly characterized,^20,21^ presenting challenges for its application in research. Despite this, the abundant production of technical lignin encourages its valorization. On the contrary, Karlsson et al. introduced a cyclic extraction technique for extracting well-defined organosolv lignin with preserved native linkages.^22−24^

Given lignin’s complexity and heterogenic structure, lignin valorization often involves depolymerization to produce mostly monomers mixed with oligomers that can be further separated to obtain more homogeneous platform chemicals. Oxidation and reduction reactions stand as prominent methods for lignin depolymerization, which can be conducted through various approaches, with or without catalysts.^7,25−27^ A comprehensive review by Lange et al. has emphasized the potential of peroxide-based methods in achieving efficient lignin depolymerization, highlighting various oxidative routes.^28^ Oxidation pathways, including autoxidation catalysis,^29^ the use of oxygen,^30,31^ hydrogen peroxide,^32−34^ or peracetic acid,^35−37^ facilitate the cleavage of interunit linkage, side-chain modification, and phenolic ring cleavage.^38^ Oxidation by peracetic acid, especially, results in cleavage of β-O-4′ bonds and ring opening of phenolic rings to form carboxylic acid.^39^ This supports the potential of peroxide-based methods for producing high-value lignin derivatives with increased carboxylic acid content.^40^ Moreover, peracetic acid can be readily produced in the reaction system by mixing acetic acid and hydrogen peroxide,^41^ making it a viable option for industrial applications.

In this work, our objective is to prepare lignin oligomers with a heightened carboxylic acid content that are suitable as precursors for polymeric materials. For this purpose, we opted for oxidation using acetic acid and hydrogen peroxide under conditions that preserved the lignin backbone. We evaluated four different lignin sources: spruce and eucalyptus kraft lignin along with spruce and birch cyclic extracted organosolv lignin. This approach allowed us to thoroughly assess how lignin’s structural variations influence its reactivity toward oxidation at the chosen conditions. We further demonstrated the potential to tailor the carboxylic acid content postoxidation.

## Experimental Section

### Materials

Spruce and eucalyptus kraft lignin from LignoBoost, Norway, spruce (*Picea abies*) sawdust, and birch wood chips were utilized in the experimental procedures. Chromium(III) acetylacetonate (Cr(AcAc_3_); 99.99%), *endo*-*N*-hydroxy-5-norbornene-2,3-dicarboximide (eHNDI; 97%), *N*,*N*-dimethylformamide (anhydrous, 99.8%), pyridine (anhydrous, 99.8%), 2-chloro-4,4,5,5-tetramethyl-1,3,2-dioxaphospholane (Cl-TMDP; 95%), CDCl_3_ (≥99.8 at. % D), sodium hydroxide (anhydrous, ≥98%, reagent grade), hydrochloric acid (1 M), [D_6_]DMSO (99.9 at. % D), and hydrogen peroxide solution (30%) were procured from Sigma-Aldrich. Sulfuric acid (>95%, analytical grade) and acetic acid (99.7%, analytical grade) were purchased from Fischer Chemicals. Ethyl acetate (≥99.9% technical grade) and ethanol absolute (99.8%, technical grade) were acquired from VWR. All chemicals and solvents were used as received with no additional purification step.

### Methods

#### Nuclear Magnetic Resonance Spectroscopy

The ^13^C, 2D HSQC (heteronuclear single quantum coherence) and ^31^P NMR (nuclear magnetic resonance) experiments were performed using a Bruker AVANCE III HD 400 MHz (Bruker Corporation, Billerica, MA, USA) instrument with a BBFO probe equipped with a *Z*-gradient coil for structural analysis at room temperature. The 2D HMBC NMR experiments were conducted using a Bruker 400 DMX (Bruker Corporation, Billerica, MA, USA) instrument equipped with a 5 mm Bruker BBI probe at room temperature. The NMR data were processed and analyzed with the use of MestReNova software (ver. 14.2.0, Mestrelab Research).

Sample preparation for 2D NMR experiments: The sample, weighing around 80 mg, was dissolved in 600 μL of [D_6_]DMSO. The HSQC spectra and parameters were set to an acquisition time of 0.0625 s, a 1.5 s relaxation delay, and 88 scans over 512 × 256 increments. Similarly, the HMBC spectra were obtained using an acquisition time of 0.2560 s, a relaxation delay of 1.5 s, and 120 scans with 1024 × 256 increments. Analysis of the data was carried out with MestReNova software, applying 1024 × 1024 data points and a 90° shifted square sine-bell window for apodization. This included Fourier transformation and phase and baseline corrections in both dimensions, employing a third-order Bernstein polynomial fit. For semiquantitative analysis of lignin interunit linkages, the C2–H signal in the aromatic ring was chosen as the internal standard.

For the ^13^C NMR experiment for oxidized lignin, 80 mg of the sample was dissolved in 600 μL of [D_6_]DMSO, and 0.01 M chromium(III) acetylacetonate (Cr(AcAc)_3_) was added as a relaxation agent to enhance spectral resolution. The ^13^C spectra were acquired with an acquisition time of 1.3631 s, a relaxation delay of 2 s, and 22,600 scans.

The quantitative ^31^P NMR analysis was conducted using a method established in prior research.^42,43^ For this, a freeze-dried sample of 30 mg was dissolved in a mixture of 100 μL of *N*,*N*-dimethylformamide and 100 μL of pyridine. This was followed by the addition of 50 μL of an internal standard (IS) solution, which contained 60 mg mL^–1^ eHNDI in pyridine and 5 mg mL^–1^ Cr(AcAc_3_) as a relaxing agent. The Cl-TMDP phosphorylation agent, amounting to 100 μL, was then introduced, and CDCl_3_ (450 μL) was added dropwise. The ^31^P NMR spectra were acquired over 256 scans with a 5 s relaxation delay. Data processing involved Fourier transformation and both phase and baseline corrections in two dimensions using a third-order Bernstein polynomial fit. The quantification of hydroxyl and carboxylic acid groups utilized the eHNDI peak at 132.2 ppm as a reference.

#### Size Exclusion Chromatography

The molecular weight and dispersity of lignin, oxidized lignin, and oxidized lignin treated with alkaline hydrolysis were determined using an SEC 1260 Infinity system (Polymer Standards Service), equipped with a PSS precolumn, a 100 Å PSS column, and a PSS GRAM 10,000 Å analytical column, all operated at 60 °C. This system incorporated both UV detector and a refractive index detector, used sequentially. For elution, DMSO containing 0.5% LiBr was used, maintaining a flow rate of 0.5 mL min^–1^. Molecular weight calibration was established using pullulan standards. For analysis, a freeze-dried sample (about 4 mg) was dissolved in 1 mL of DMSO with 0.5 wt % LiBr. These solutions were then filtered using a syringe equipped with a 0.45 mm PTFE filter, ensuring sample solubility in the DMSO solution.

### Procedures

#### Extraction of Wood

Extraction of spruce and birch samples was performed using an accelerated solvent extractor (ASE) 350, operated with Chromeleon 7.2.10 software (Dionex, Sunnyvale, CA, USA). This process was based on the methodologies described in publication reported by Karlsson et al.^23,24^ For both spruce and birch, the extraction was carried out at a controlled temperature of 140 °C.

#### Lignin Oxidation in Acidic Condition

The lignin oxidation process was performed based on the protocol established by Park et al.^36^ The oxidation reaction was performed using a mixture of acetic acid and hydrogen peroxide in a ratio of 80:20 (v/v). This solution was allowed to stabilize for 1 h. In each experiment, 600 mg of lignin was added to a round-bottom flask, combined with 18 mL of the acetic acid–hydrogen peroxide solution, and the setup included a condenser to prevent solvent evaporation. The oxidation reaction was conducted at 80 °C for 60 min for various lignin types: LignoBoost spruce kraft lignin (SKL), LignoBoost eucalyptus kraft lignin (EKL), spruce cyclic extracted organosolv lignin (SCOL), and birch cyclic extracted organosolv lignin (BCOL). Following the reaction, the mixtures were cooled and diluted with 10-fold volume in Milli-Q water, followed by centrifugation at 4800 rpm for 1 h using a ROTINA 420R centrifuge. The resulting precipitate was washed with acidic water (pH 2) and freeze-dried for 24 h.

The remaining lignin in the water phase was extracted using ethyl acetate (EtOAc) in a 1:1 volume ratio, repeated twice to ensure thorough extraction. The oxidized lignin, now in EtOAc, was then evaporated under reduced pressure, which resulted in an oily product. This product was dissolved again in a mixture of EtOAc and Milli-Q water, followed by a second evaporation to remove the EtOAc. Finally, the mixture underwent freeze-drying for 24 h to obtain oxidized lignin in powder form. The remaining lignin in the water phase that was not extracted by EtOAc was subsequently treated with CaCl_2_, then centrifuged and freeze-dried to obtain the water-soluble fraction in powder form.

#### Alkaline Hydrolysis Treatment

The EtOAc soluble oxidized lignin samples were treated with 0.1 M NaOH at 60 °C for 3 h. The pH was then adjusted to 1 using 1 M HCl, which induced oxidized lignin precipitation. After 30 min of centrifugation at 4800 rpm, the precipitate was washed with acidic water (pH 2) and freeze-dried for 24 h. The lignin retained in the aqueous phase was extracted with EtOAc in a 1:1 volume ratio, evaporated under reduced pressure, and subsequently freeze-dried for 24 h after redissolving and evaporating in EtOAc and Milli-Q water as described earlier.

## Results and Discussion

### Lignin Oxidation in Acidic Condition

The mixture of 80% acetic acid and 20% hydrogen peroxide forms peracetic acid. The reaction condition used in this work was chosen based on what was found to be optimal from the literature.^36^ In this system, the hydroxonium cation (HO^+^), a key oxidizing agent, is proposed to effectively target electron-dense regions of lignin-forming carboxylic acid groups and acid esters.^44^

Following the oxidation reaction, dilution of the system with water yielded partitioning of the oxidized lignin products into three distinct fractions. First, a precipitated fraction was formed. Second, a more water-soluble fraction was extracted using ethyl acetate (EtOAc), subsequently termed the EtOAc-soluble fraction. Finally, the remaining water-soluble fraction, which was not extracted with EtOAc, was extracted using Ca^2+^ ions and subsequently referred to as the Ca^2+^ ion-extracted water-soluble fraction. The yields of these oxidized lignin products are detailed in Table 1. Overall yields of precipitated and EtOAc-soluble fraction were between 59 and 85%, with the highest yields observed for the lignin from softwoods. A portion of the water-soluble fraction was successfully recovered by extraction with Ca^2+^ ions; however, it was not quantified due to weight interference from the ions. This result suggests that hardwood lignins were more prone to degradation than the softwood lignins under oxidation conditions.

**Table 1:** Yield of Precipitated and EtOAc-Soluble Fractions of Oxidized Lignin Samples

sample	precipitated fraction		EtOAc-soluble fraction		water-soluble fraction	
	abbreviation	yield (%)^a^	abbreviation	yield (%)^b^	abbreviation	yield (%)^c^
OSKL	OSKL_precip	13	OSKL_EtOAc	51	OSKL_water	36
OEKL	OEKL_precip	1	OEKL_EtOAc	58	OEKL_water	41
OSCOL	OSCOL_precip	32	OSCOL_EtOAc	53	OSCOL_water	15
OBCOL	OBCOL_precip	20	OBCOL_EtOAc	63	OBCOL_water	17

a The yield of precipitated fraction was calculated based on the dry mass of precipitated fraction after the workup step of oxidation reaction, compared to the initial mass of lignin before reaction.

b The yield of EtOAc-soluble fraction was calculated based on the dry mass of EtOAc-soluble fraction after EtOAc extraction of water phase from oxidation reaction, compared to the initial mass of lignin before reaction.

c Theoretical yield of water-soluble fraction.

It was noted that the quantity of precipitated oxidized lignin was less than that of EtOAc-soluble oxidized lignin. This difference can be attributed to the oxidation reaction that increased the carboxylic acid content and enhanced the hydrophilicity of lignin. Consequently, the oxidized lignin became more soluble in water, favoring the formation of the EtOAc-soluble fraction. Additionally, oxidized lignin from cyclic extracted organosolv lignin of spruce and birch had a higher yield for the precipitated fraction compared to that from spruce and eucalyptus kraft lignin. This was likely due to the fact that kraft lignins become relatively more hydrophilic upon oxidation and was supported by the observation that the recovery yields of the oxidized cyclic extracted organosolv lignins were significantly higher than those of the kraft equivalents. Furthermore, the heterogeneous nature in lignin significantly influenced its reactivity. Even within the same lignin type, heterogeneity in structure, molecular weight, and functionality has been documented^20,21^ and certainly reflected in the reactivity differences reported here. A comprehensive explanation for these observations was derived from the structural analyses and molecular weight trends discussed in the following sections.

### Analysis of Structural Changes

**Figure 1:**
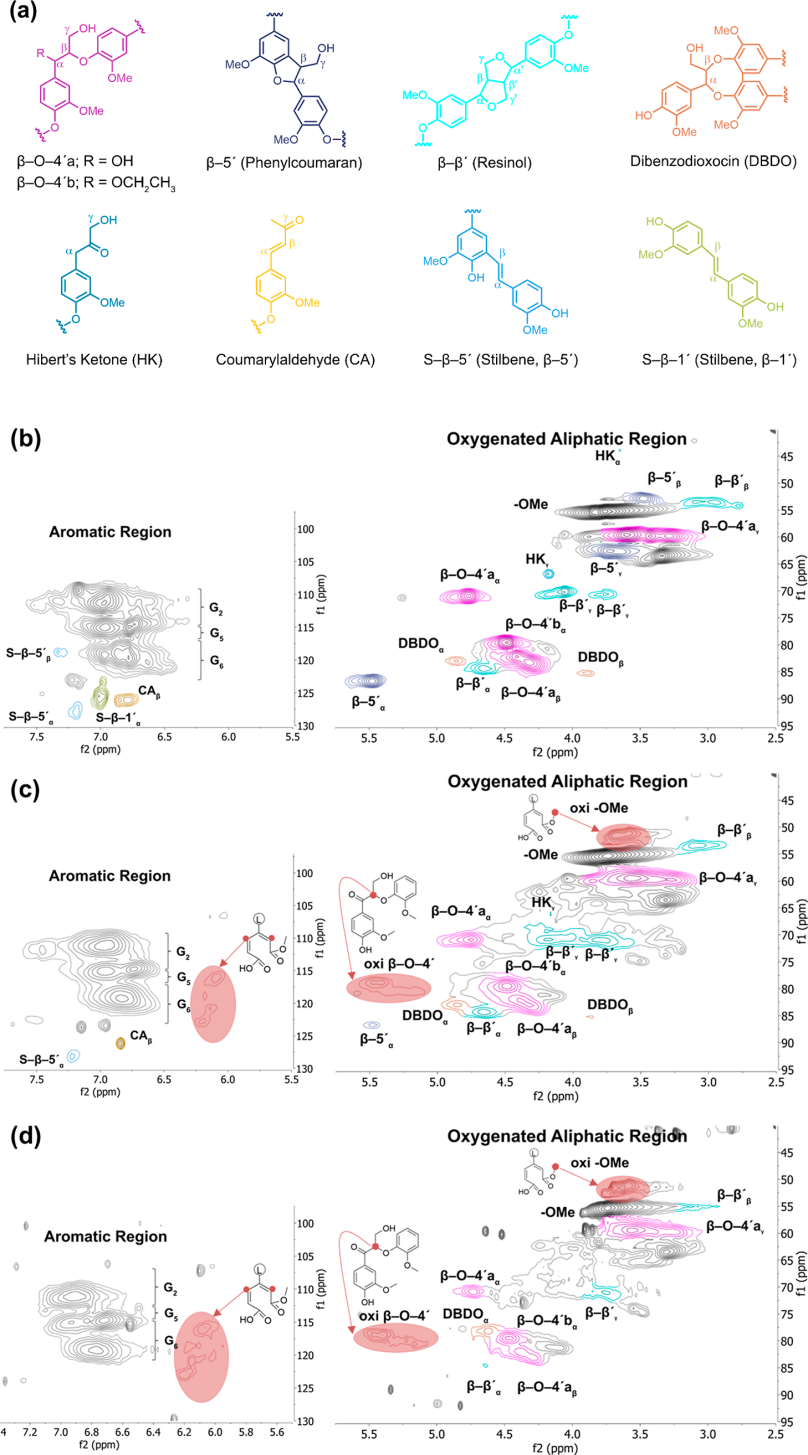
(a) Common interunit linkages in lignin; 2D HSQC NMR spectra of (b) reference spruce cyclic extracted organosolv lignin; (c) oxidized spruce cyclic extracted organosolv lignin precipitated fraction (OSCOL_precip), and (d) oxidized spruce cyclic extracted organosolv lignin EtOAc-soluble fraction (OSCOL_EtOAc), with f1 for ^13^C and f2 for ^1^H.

The investigation of the structural transformations of oxidized lignin samples employed 2D HSQC and 2D HMBC NMR experiments. A key alteration in the lignin structure was the ring opening of phenolic rings to form muconic acid and methyl ester as end groups in the lignin oligomers. The 2D HSQC spectra, illustrated in Figure 1, reveal the structural difference of oxidized spruce cyclic extracted organosolv lignin. This discussion focuses on the precipitated and EtOAc-soluble fractions. Predominantly, Figure 1 highlights the appearance of new signals at δ = 3.68/51.5 ppm and δ = 3.70/51.8 ppm for both precipitated and EtOAc-soluble fractions, respectively, assigned to methyl ester linked to the muconic acid, a product of phenolic ring opening. Additionally, the aromatic region of the spectra displays a new signal cluster that appeared around δ = 6/115–123 ppm, corresponding to double bonds resulting from the phenolic ring’s opening. Another significant observation was a signal at δ = 5.4/78.9 ppm in both ^1^H/^13^C signals, corresponding to β-O-4′ linkages connected to the oxidized conjugated side chain in the α position. Noteworthy is the preservation of the native interunit linkages as a testimony to the mildness and selective nature of the oxidation conditions.

It was also noteworthy that the precipitated and EtOAc-soluble fractions of oxidized spruce cyclic extracted organosolv lignin exhibited interesting structural differences, as depicted in Figure 1. Specifically, the OSCOL_precip fraction (Figure 1c) contained the common interunits such as β-O-4′, β-5′, and β-β′ bonds compared to the OSCOL_EtOAc fraction (Figure 1d), which was devoid of β-5′ and β-β′ bonds. This finding indicated the presence of two distinct lignin populations within the native-like cyclic extracted organosolv lignin: one that was more reactive and richer in β-O-4′ bonds, and the other still dominated by β-O-4′ bonds but also contained less reactive bonds such as β-5′ and β-β′. This distinction in lignin populations aligned with findings by Lawoko et al.,^45^ where similar structurally distinct lignin isolated from plant cell walls. Further support for this observation was recently derived from MALDI studies by Karlsson et al.,^46^ identifying octameric structures in the spruce cyclic organosolv lignin that were back-tracked to β-O-4 oligomers.

Figures S1–S8 present the NMR analysis of the other lignin types. The same new signals linked to the oxidation reactions in spruce kraft lignin, eucalyptus kraft lignin, and birch cyclic extracted organosolv lignin are observed, illustrating a consistent trend across the lignin types. This trend mirrored the observations previously discussed for oxidized spruce cyclic extracted organosolv lignin. Particularly, in hardwood lignins (as depicted in Figures S4–S8), an interesting shift in the S/G unit ratio revealed their differential in reactivity. More specifically, the EtOAc-soluble fraction of oxidized eucalyptus kraft lignin (OEKL_EtOAc) exhibited an S/G ratio of 1.13. This represented a decrease from the 2.72 S/G ratio observed in the reference pristine lignin. Similarly, analyses of both precipitated and EtOAc-soluble fractions of oxidized birch cyclic extracted organosolv lignin (OBCOL_precip and OBCOL_EtOAc) revealed S/G ratios ranging between 0.5 and 0.6. This result indicated a significant decline from the 4.33 S/G ratio found in their respective reference pristine lignin. These findings demonstrated an increased presence of G units in hardwood lignin following oxidation, suggesting that S units are more prone to oxidation and subsequent ring opening. The higher reactivity of hardwood lignin was recently explored by Andriani et al.,^47^ who proposed that the molecular structure of S-rich lignin enhanced the accessibility of reactive sites due to the greater exposure of hydroxyl groups in S units, as opposed to the more compact and less accessible structure in G units.

The 2D HMBC experiments, illustrated in Figures S10 and S11, validate the phenolic ring opening in both spruce kraft lignin and spruce cyclic extracted organosolv lignin, as evidenced by the correlation between the proton in the methyl ester and the carbonyl carbon. Complementary ^13^C NMR experiments (Figures S12 and S13) further confirm the formation of new methoxyl groups associated with the methyl ester linked to the muconic acid moiety introduced in the lignin. These ^13^C NMR studies were conducted on selected samples, including spruce kraft lignin and spruce cyclic extracted organosolv lignin. The quantitative analysis utilized ^13^C NMR spectra to measure the methoxy groups in the oxidized kraft spruce lignin (soluble in EtOAc). This analysis focused on the aromatic region’s carbon area integral within 105–156 ppm, the methoxyl group in the phenolic ring at 54–58 ppm, and the methyl ester linked to the muconic acid moiety at 50–54 ppm. The results revealed that the EtOAc-soluble fraction of oxidized kraft spruce lignin contained 0.7 methoxyl group per phenolic ring and 0.3 methyl ester linked to the muconic acid moiety, providing clear evidence of the ring opening of the phenolic structure during oxidation.

**Figure 2:**
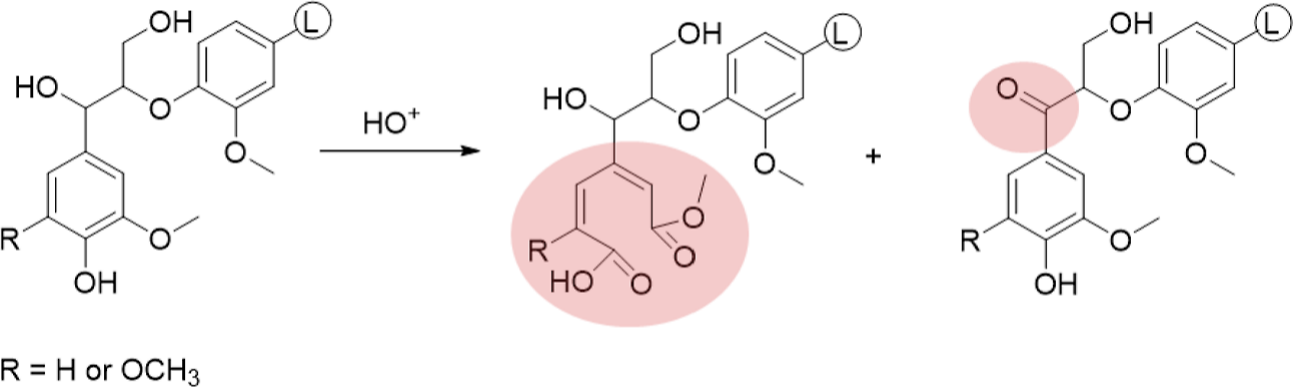
Oxidation reaction induced by peracetic acid.

Therefore, the structural changes observed in 2D HSQC spectra, prompted by oxidation with peracetic acid—a mixture of acetic acid and hydrogen peroxide—resulted in the formation of two distinct products: muconic acid ester and a ketone positioned at the α site of the conjugated side chain, as represented in Figure 2.

### Mechanistic Discussion

Initially, acetic acid and hydrogen peroxide in the reaction mixture will form peroxyacetic acid. Both peroxides, in turn, undergo protonation to form the hydroxyl cation (HO^+^), an electrophile that has been proposed to trigger several reactions, which include the introduction of hydroxyl groups into aromatic rings, displacement of phenyl propane side chains, oxidation of benzylic hydroxyls, epoxidation of olefin structures, oxidative demethylation, and aromatic ring-opening reactions.^48^ Based on the NMR analysis, the most prominent reactions of relevance to the oxidized lignin isolated from our reaction system were ring-opening reaction forming muconic acid moieties and methyl esters and the oxidation of benzylic hydroxyls in β-O-4′ substructures to ketones, Figure 2.

### Hydroxy Functionality Content

The hydroxy functionality content in reference pristine and oxidized lignin samples was quantified using ^31^P NMR spectroscopy, following established protocols.^42,43^ The hydroxy functionality of the Ca^2+^ ion-extracted water-soluble fraction could not be determined due to interference from the Ca^2+^ ions affecting the measurement of hydroxyl groups. Spectra can be found in the Supporting Information (Figures S14–S29). The precipitated and EtOAc-soluble fractions of oxidized lignin showed an increase in carboxylic acid content when compared to the reference pristine lignin. However, the rise in carboxylic acid content was less pronounced in the precipitated fraction compared to that in the EtOAc-soluble fraction (Table 2). As described earlier, this discrepancy was ascribed to the existence of two populations in the reference pristine lignin samples, distinct by their structure, which dictated the proneness to reactivity during oxidation. The EtOAc-soluble fraction that originated from the population was more susceptible to cleavage and ring opening of phenolic rings during oxidation. This observation was supported by the decrease in signal intensity of C2 aromatic observed in HSQC NMR analysis. This decrease was compensated by the appearance of new signals assigned to double bonds in muconic acid moieties, as discussed earlier.

**Table 2:** Quantification of the Lignin Functional Groups

samples	aliphatic—OH (mmol/g)^a^	phenolic—OH (mmol/g)^a^	carboxylic acid (mmol/g)^a^
SKL	2.5	3.9	0.6
OSKL_precip	1.8	1.4	1.7
OSKL_EtOAc	1.3	1.6	3.5
EKL	1.6	4.8	0.5
OEKL_EtOAc	1.1	1.5	3.5
SCOL	4.4	1.5	0.1
OSCOL_precip	3.0	1.3	0.9
OSCOL_EtOAc	2.9	1.7	2.7
BCOL	4.0	1.4	0.2
OBCOL_precip	2.1	0.9	1.0
OBCOL_EtOAc	1.9	1.3	3.5

a Data was calculated based on ^31^P NMR analysis.

Table 2 shows that for OSKL_EtOAc and OEKL_EtOAc, both aliphatic and phenolic hydroxyls decreased, while carboxylic acid content increased significantly. In contrast, OSCOL_EtOAc and OBCOL_EtOAc showed a similar trend in hydroxy functionality content, except for phenolic hydroxyls. Notably, both OSCOL_EtOAc and OBCOL_EtOAc showed a miniscule increase in phenolic hydroxyl content, which could result from hydroxylation of aromatic rings or partial hydrolysis of β-O-4′ bonds.^24^

Interestingly, the increase in the carboxylic acid content was significantly more than the decrease in the phenolic hydroxyl content, Table 2, suggesting that ring-opening reactions were not confined to phenolic end groups but also occurred on 4-O-etherified aromatics. This observation aligned with a high content of methyl esters identified by 2D HSQC NMR analyses discussed earlier. All EtOAc-soluble fractions of oxidized lignin samples from different sources exhibited a similar increase in the carboxylic acid content of approximately 3 mmol/g. These results were comparable with the carboxylic acid content of lignin obtained through carboxymethylation modification, as reported in our previous research.^47^

### Enhancing the Carboxylic Content of Oxidized Lignin by Alkaline Hydrolysis

The formation of methyl esters in the ring-opening reactions of phenolic rings presented an opportunity to enhance the carboxylic acid groups in the oxidized lignin samples. This increase was evident as the oxidized lignin displayed new methoxyl groups (methyl esters), resulting from the ring-opening reaction of aromatics in lignin. The methyl esters formed could be hydrolyzed to carboxylic acids and methanol, thus, further increasing the carboxylic acid groups in the oxidized lignin. To maintain control over the reaction, an alkaline hydrolysis treatment was conducted under mild conditions.

**Figure 3:**
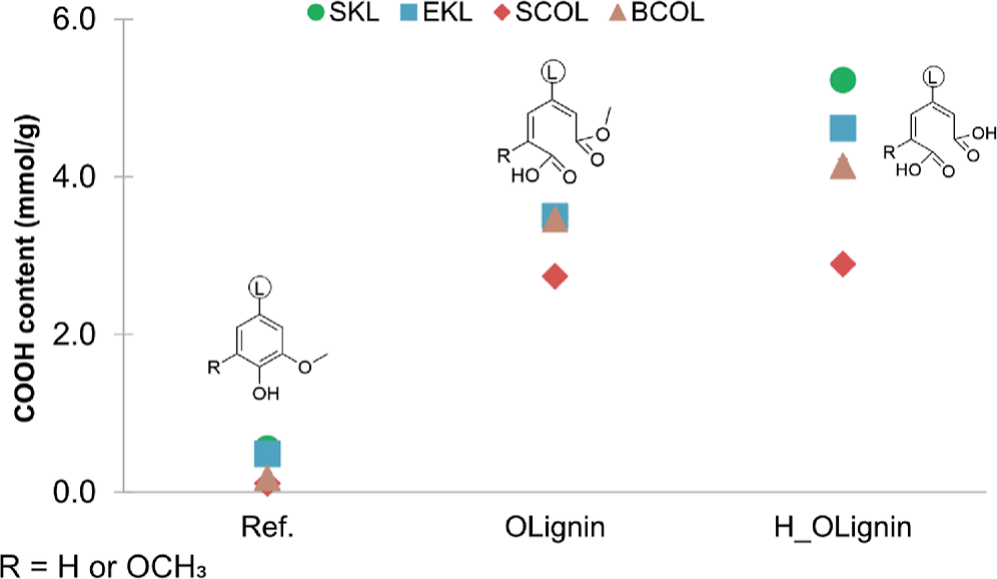
Effect of alkaline hydrolysis treatment on the carboxylic acid content of EtOAc-soluble oxidized lignin samples.

In this work, alkaline hydrolysis treatment was performed on EtOAc-soluble oxidized lignin samples. As previously, the products were recovered as a precipitated and EtOAc-soluble fraction. The overall yield from this treatment ranged between 67 and 84%, as detailed in Table S3. However, the precipitated part only accounted for about 7.5–30% of the yield. We, therefore, focused on the analysis of the EtOAc-soluble fraction. Figure 3 shows a significant increase in the carboxylic acid content in most samples following the treatment. These findings demonstrated the successful conversion of methyl ester groups (resulting from the formation of new methoxyl groups) into carboxylic acid groups. Specifically, OSKL_EtOAc exhibited the highest increase in the carboxylic acid content of 1.7 mmol/g, followed by OEKL_EtOAc with an increase of 1.1 mmol/g. Interestingly, in the case of oxidized lignin derived from cyclic-extracted organosolv lignin, OBCOL_EtOAc displayed a higher increase in carboxylic acid content compared to OSCOL_EtOAc, with increments of 0.7 and 0.2 mmol/g, respectively.

The higher increase observed in birch-derived oxidized lignin compared with spruce-derived lignin was attributed to the higher presence of methyl esters derived from the ring opening of syringyl units. This observation aligned with the earlier discussed 2D NMR results in support of higher reactivity of syringyl units toward ring-opening reactions when contrasted with guaiacyl units. Further support for the conversion from methyl ester to carboxylic acid groups was obtained through the 2D HSQC spectra which showed significant decreases of signals corresponding to methyl esters at ^1^H/^13^C resonances of δ = 3.72/52.1 ppm in 2D HSQC spectra, as depicted in, e.g., the comparison of Figure S5 with S9. These results highlighted how easily the carboxylic acid content in oxidized lignin samples can be tailored through alkaline hydrolysis treatment, thus, enhancing the versatility of this oxidized lignin as a precursor for various applications.

### Molecular Weight and Dispersity

The results on the molecular weight and dispersity of reference pristine, oxidized lignin, and hydrolyzed oxidized lignin samples, as presented in Table 3 and Figures S30–S33, provide further validation of the discussion in the previous section on the impact of the oxidation reaction.

**Table 3:** Molecular Weight and Dispersity of the Reference, Oxidized Lignin, and Hydrolyzed Oxidized Lignin Samples

samples	*M*_n_^a^	*M*_w_^a^	*D̵*^a^
SKL	2092	9404	4.50
OSKL_precip	2889	6158	2.13
OSKL_EtOAc	1043	1938	1.86
OSKL_Ca^2+^	3932	10475	2.66
H_OSKL_EtOAc	399	938	2.35
EKL	1105	2404	2.18
OEKL_precip	1104	2181	1.98
OEKL_EtOAc	362	1067	2.95
OEKL_Ca^2+^	4042	8376	2.07
H_OEKL_EtOAc	399	938	2.35
SCOL	2426	13641	5.62
OSCOL_precip	2497	5123	2.05
OSCOL_EtOAc	901	1471	1.63
H_OSCOL_EtOAc	441	1410	3.20
BCOL	2347	10819	4.61
OBCOL_precip	2192	4881	2.23
OBCOL_EtOAc	819	1379	1.68
H_OBCOL_EtOAc	358	958	2.67

a Data were obtained by SEC–DMSO.

Across all oxidized lignin samples, the precipitated fraction exhibited a higher number average molecular weight (*M*_n_) compared to the reference lignin samples, while the EtOAc-soluble oxidized lignin showed lower *M*_n_ values. This discrepancy can be ascribed to the heterogeneous nature of lignin with the lower molecular weight fractions likely more prone to oxidation, as manifested by the NMR analyses. Interestingly, the precipitated, EtOAc-soluble, and Ca^2+^ ion-extracted water-soluble oxidized lignin displayed lower dispersity compared to the reference pristine lignin. Combined, the results of the *M*_n_ and dispersity suggest two things; (i) the oxidation reaction, in some cases, led to some level of lignin degradation, further contributing to the reduction in the molecular weight, particularly in the EtOAc-soluble fraction, and (ii) the changes in lignin properties through oxidation had a positive effect on the refining of lignin with respect to the molecular weight.

According to the SEC data (Table 3), the water-soluble fraction, extracted with Ca^2+^ ions, exhibited a higher *M*_n_. Although this was not initially expected, it turned out to be logical since EtOAc has previously been shown to extract lower molecular weight lignin,^49^ leaving larger molecules in the water phase. These larger molecules were observed to be in the same molecular weight range as the precipitated fraction but differed from the latter by a higher carboxylic acid content, as observed by NMR (Figures S34 and S35). This difference likely explained their retention in the water phase caused by higher hydrophilicity. In contrast, the molecular weight of the recovered EtOAc-soluble oxidized lignin samples was moderate, constituting oligomeric structures. This evidenced the successful oxidation of lignin while still preserving a significant part of the lignin backbone.

Furthermore, the analysis of the alkaline-treated oxidized lignins indicated that some hydrolysis of lignin bonds occurred in addition to the hydrolysis of methyl esters. In support of lignin hydrolysis, a further review of the 2D NMR of the EtOAc-soluble and alkaline-treated equivalent (contrast S5 with S9) shows the disappearance of signals assigned to benzylic carbonyls in β-O-4′ substructures at δ = 5.4/78.9 ppm. Thus, the tailoring of the reduction in the molecular weight of oxidized lignin was achieved simultaneously with the increase in carboxylic content by the post alkaline treatment. Overall, from the NMR and SEC, the number of carboxylic acid groups per macromolecule (designated *n*) can be estimated using
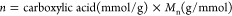
1where the carboxylic acid content was calculated from ^31^P NMR data and *M*_n_ is the number average molecular weight obtained from SEC. According to the calculation, the precipitated fractions of oxidized lignin samples contained 2–5 carboxylic acid groups per macromolecule, while the EtOAc-soluble fraction of oxidized lignin samples contained 3–4 carboxylic acid groups per macromolecule. These results rendered the macromolecules multifunctional with respect to carboxyl functionality.

The structure of these oxidized lignin samples represents novel precursors for advanced applications such as thermosets based on renewable aromatics, where the carboxylic acid groups can be used as chemical handles in cross-linking reactions. Thermosets made from lignin, a natural polymer found in wood, have shown great potential.^11^ Our work opens the door for further advancements in making these thermosets by introducing versatile chemical connectors that could make these materials recyclable when they are no longer needed. Studies to explore these possibilities are currently in progress.

## Conclusions

We investigated the impact of oxidation reactions on the molecular structure of various lignin types, including spruce kraft lignin, eucalyptus kraft lignin, spruce cyclic extracted organosolv lignin, and birch cyclic extracted organosolv lignin. The differences between kraft lignins and cyclic extracted organosolv lignin are significant. One major difference is that kraft lignins have carbon–carbon bonds between monomers due to condensation reactions, while cyclic organosolv lignins are more native-like and have a high content of aryl ether bonds and aliphatic hydroxyls. Additionally, hardwood lignins have a higher abundance of syringyl units in contrast with softwood lignins, which are entirely composed of guaiacyl units. This study, therefore, evaluated the responsiveness of these molecularly distinct lignin structures toward oxidation by peroxides in acidic conditions. A comprehensive analysis of postoxidation revealed alterations in the lignin structure, notably through the ring opening of phenolic structures that led to muconic acid generation.

This significant transformation was evidenced by the appearance of new NMR signals corresponding to muconic acid moieties and methyl esters, which is a direct consequence of these ring-opening reactions. In addition, the oxidation facilitated the oxidation of benzylic hydroxyls, as confirmed by the appearance of a new NMR signal, assigned benzylic carbonyls in β-O-4′ substructures. Notably, the structure of the lignin macromolecule was mostly preserved in the recovered oxidized lignin fractions. Accordingly, the oxidized lignin oligomers were shown to be multifunctional in carboxylic acid functionality. Each macromolecule was estimated to have between 2 and 5 carboxylic acid groups.

The incorporation of a postoxidation alkaline hydrolysis treatment built upon this effect amplifies the carboxylic acid content and offers a promising avenue for tailoring lignin structures to meet specific application requirements. Moreover, the results revealed that the recovered oxidized lignins had lower dispersity than the pristine lignins. The study offers deep insights into how oxidation reactions can be harnessed to modify various lignin structures, paving the way for the development of tailored lignin-based materials.

## Supporting Information Available

2D HSQC NMR spectra, 2D HMBC NMR spectra of EtOAc-soluble fraction of oxidized spruce kraft lignin and oxidized spruce cyclic extracted organosolv lignin, ^13^C NMR spectra of EtOAc-soluble fraction of oxidized spruce kraft lignin and oxidized spruce cyclic extracted organosolv lignin, assignment of ^31^P NMR chemical shifts, quantification of lignin functional groups, ^31^P NMR spectra, SEC–DMSO results, and yield of both precipitated and EtOAc-soluble fraction of oxidized lignin samples after alkaline hydrolysis treatment (PDF)
