# Supplementary material for: Oxidative Carboxylation of Lignin: Exploring Reactivity of Different Lignin Types
Source: Biomacromolecules. 2024 Jun 13;25(7):4246–54. doi: 10.1021/acs.biomac.4c00326 (PMC11238328; doi:10.1021/acs.biomac.4c00326)
Supplement: Supplementary file 1 — bm4c00326_si_001.pdf [file bm4c00326_si_001.pdf]

Supporting Information

Oxidative Carboxylation of Lignin: Exploring Reactivity of Different Lignin Types

Fika Andriani,[1] *and Martin Lawoko*[*1,2]

[1] Division of Wood Chemistry and Pulp technology, Department of Fiber and Polymer Technology, School of Chemistry, Biotechnology and Health, KTH Royal Institute of Technology, SE-100 44 Stockholm, Sweden

[2] Wallenberg Wood Science Center, Department of Fiber and Polymer Technology, School of Chemistry, Biotechnology and Health, KTH Royal Institute of Technology, SE-100 44 Stockholm, Sweden

*Corresponding author, e-mail: lawoko@kth.se

- Number of pages: 24
- Number of figures: 35
- Number of tables: 3

Table of Contents

1. 2D HSQC NMR Spectra S4

Figure S1. 2D HSQC NMR spectra of reference spruce kraft lignin S4

Figure S2. 2D HSQC NMR spectra of oxidized spruce kraft lignin of precipitated fraction (OSKL_precip) S4

Figure S3. 2D HSQC NMR spectra of oxidized spruce kraft lignin of EtOAc-soluble fraction (OSKL_EtOAc) S5

Figure S4. 2D HSQC NMR spectra of reference eucalyptus kraft lignin S5

Figure S5. 2D HSQC NMR spectra of oxidized eucalyptus kraft lignin of EtOAc-soluble fraction (OEKL_EtOAc) S6

Figure S6. 2D HSQC NMR spectra of reference birch cyclic extracted organosolv lignin S6

Figure S7. 2D HSQC NMR spectra of oxidized birch cyclic extracted organosolv lignin of precipitated fraction (OBCOL_precip) S7

Figure S8. 2D HSQC NMR spectra of oxidized birch cyclic extracted organosolv lignin of EtOAc-soluble fraction (OBCOL_EtOAc) S7

Figure S9. 2D HSQC NMR spectra of oxidized eucalyptus kraft lignin of EtOAc-soluble fraction (OBCOL_EtOAc) after alkaline hydrolysis S8

2. 2D HMBC NMR Spectra S9

Figure S10. 2D HMBC NMR spectra of oxidized spruce kraft lignin of EtOAc-soluble fraction (OSKL_EtOAc) S9

Figure S11. 2D HMBC NMR spectra of oxidized spruce cyclic extracted organosolv lignin of EtOAc-soluble fraction (OSCOL_EtOAc) S10

3. ^{13}C NMR Spectra S11

Figure S12. 13C NMR spectra of oxidized spruce kraft lignin of EtOAc-soluble fraction (OSKL_EtOAc) S11

Figure S13. 13C NMR spectra of oxidized spruce cyclic extracted organosolv lignin of EtOAc-soluble fraction (OSCOL_EtOAc) S11

4. ^{31}P NMR S12

Table S1. The chemical shifts of 31P NMR, with water peak from Cl-TMDP at 132.2 ppm as reference S12

Table S2. Quantification of lignin functional groups S12

Figure S14. ^{31}P NMR spectra of reference spruce kraft lignin (SKL) S13

Figure S15. ^{31}P NMR spectra of oxidized spruce kraft lignin of precipitated fraction (OSKL_precip) S13

Figure S16. ^{31}P NMR spectra of oxidized spruce kraft lignin of EtOAc-soluble fraction (OSKL_EtOAc) S14

Figure S17. ^{31}P NMR spectra of oxidized spruce kraft lignin of EtOAc-soluble fraction (OSKL_EtOAc) after alkaline hydrolysis S14

Figure S18. ^{31}P NMR spectra of reference eucalyptus kraft lignin (EKL) S15

Figure S19. ^{31}P NMR spectra of oxidized eucalyptus kraft lignin of precipitated fraction (OEKL_precip) S15

Figure S20. ^{31}P NMR spectra of oxidized eucalyptus kraft lignin of EtOAc-soluble fraction (OEKL_EtOAc) S16
Figure S21. ^{31}P NMR spectra of oxidized eucalyptus kraft lignin of EtOAc-soluble fraction (OEKL_EtOAc) after alkaline hydrolysis S16
Figure S22. ^{31}P NMR spectra of reference spruce cyclic extracted organosolv lignin (SCOL) S17
Figure S23. ^{31}P NMR spectra of oxidized spruce cyclic extracted organosolv lignin of precipitated fraction (OSCOL_precip) S17
Figure S24. ^{31}P NMR spectra of oxidized spruce cyclic extracted organosolv lignin of EtOAc-soluble fraction (OSCOL_EtOAc) S18
Figure S25. ^{31}P NMR spectra of oxidized spruce cyclic extracted organosolv lignin of EtOAc-soluble fraction (OSCOL_EtOAc) after alkaline hydrolysis S18
Figure S26. ^{31}P NMR spectra of reference birch cyclic extracted organosolv lignin (BCOL) S19
Figure S27. ^{31}P NMR spectra of oxidized birch cyclic extracted organosolv lignin of precipitated fraction (OBCOL_precip) S19
Figure S28. ^{31}P NMR spectra of oxidized birch cyclic extracted organosolv lignin of EtOAc-soluble fraction (OBCOL_EtOAc) S20
Figure S29. ^{31}P NMR spectra of oxidized birch cyclic extracted organosolv lignin of EtOAc-soluble fraction (OBCOL_EtOAc) after alkaline hydrolysis S20

5. SEC S21

Figure S30. (a) Elution volume; (b) molecular weight of SKL, OSKL_precip, OSKL_EtOAc and H_OSKL_EtOAc S21
Figure S31. (a) Elution volume; (b) molecular weight of EKL, OEKL_precip, OEKL_EtOAc and H_OEKL_EtOAc S21
Figure S32. (a) Elution volume; (b) molecular weight of SCOL, OSCOL_precip, OSCOL_EtOAc and H_OSCOL_EtOAc S22
Figure S33. (a) Elution volume; (b) molecular weight of BCOL, OBCOL_precip, OBCOL_EtOAc and H_OBCOL_EtOAc S22

6. Yield of Oxidized Lignin after Alkaline Hydrolysis Treatment S23

Table S3. Yield of both precipitated and EtOAc-soluble fraction of oxidized lignin samples after alkaline hydrolysis treatment S23

7. 2D HSQC NMR of Ca^{2+} Ion-Extracted Water-Soluble Fraction of Oxidized Lignin S23

Figure S34. 2D HSQC NMR spectra of oxidized spruce kraft lignin of Ca^{2+} ion-extracted water-soluble S24
Figure S35. 2D HSQC NMR spectra of oxidized eucalyptus kraft lignin of Ca^{2+} ion-extracted water-soluble S24

1. 2D HSQC NMR Spectra

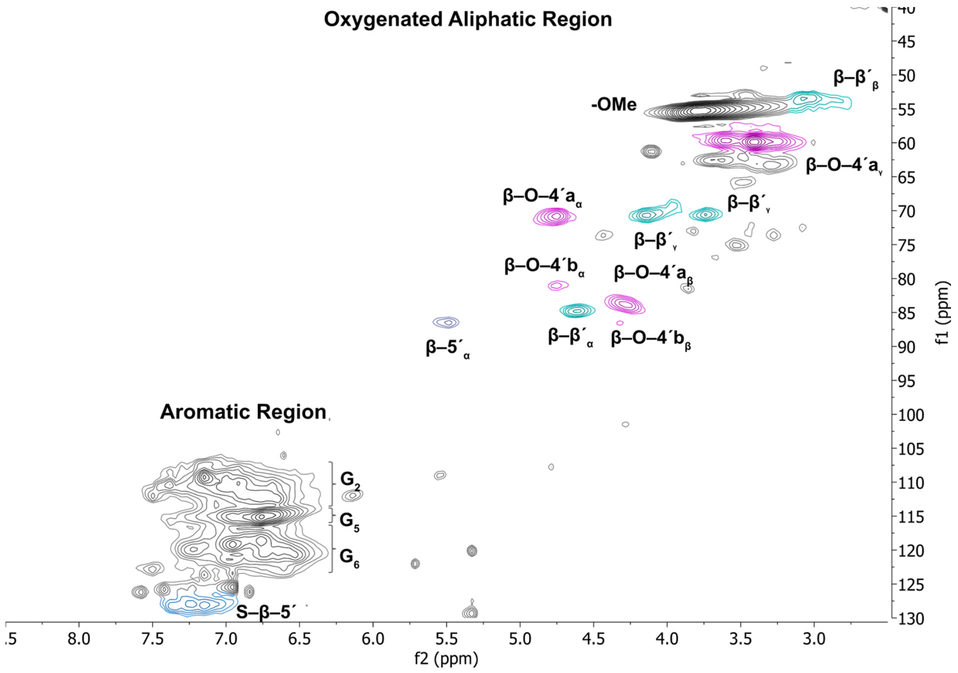

Figure S1. 2D HSQC NMR spectra of reference spruce kraft lignin, with f1 for ^{13}C and f2 for ^{1}H.

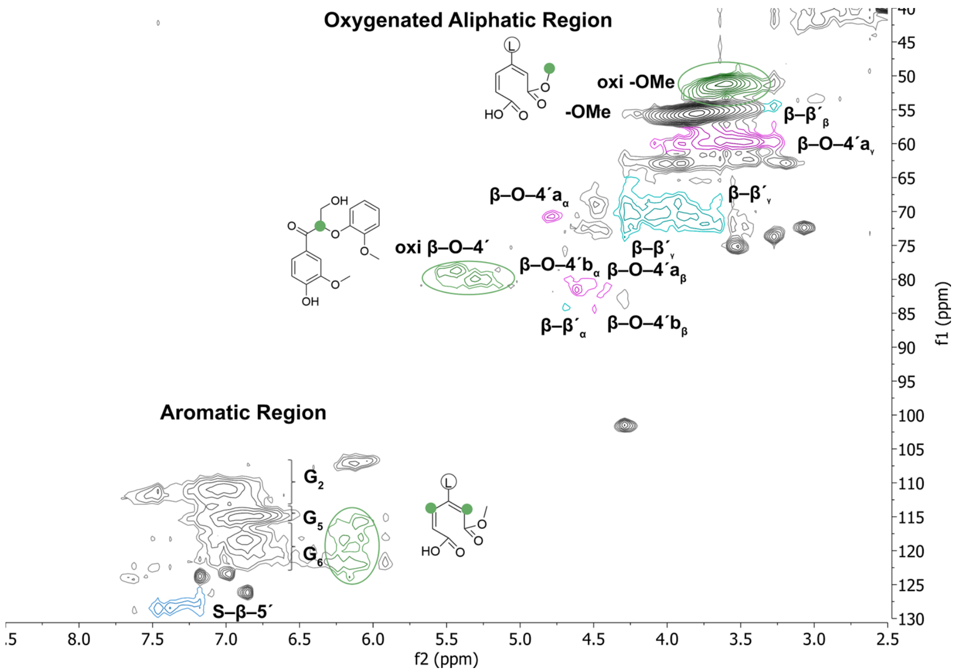

Figure S2. 2D HSQC NMR spectra of oxidized spruce kraft lignin of precipitated fraction (OSKL_precip), with f1 for ^{13}C and f2 for ^{1}H.

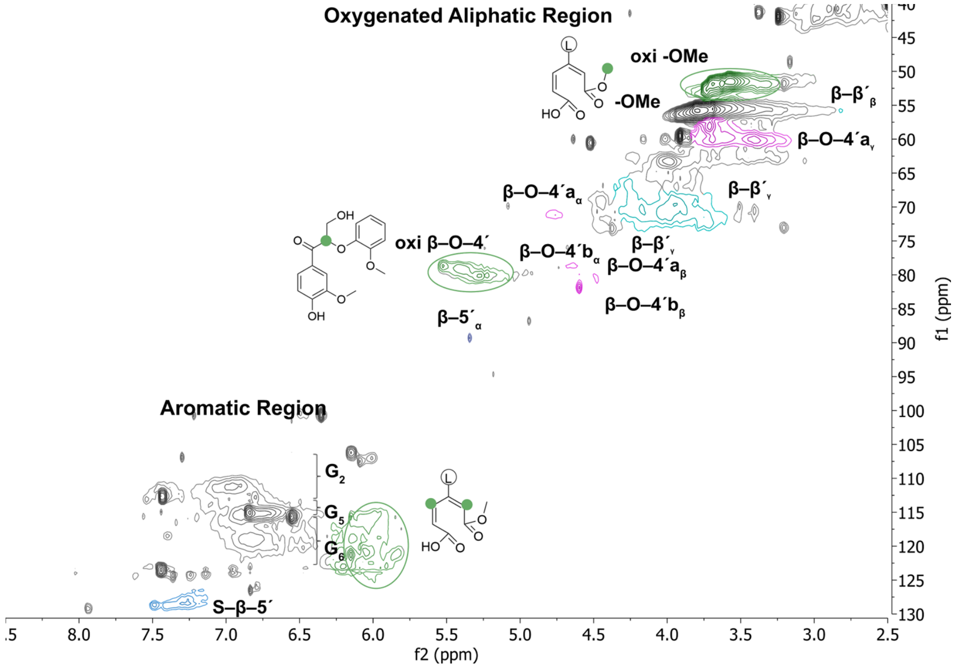

Figure S3. 2D HSQC NMR spectra of oxidized spruce kraft lignin of EtOAc-soluble fraction (OSKL_EtOAc), with f1 for ^{13}C and f2 for ^{1}H.

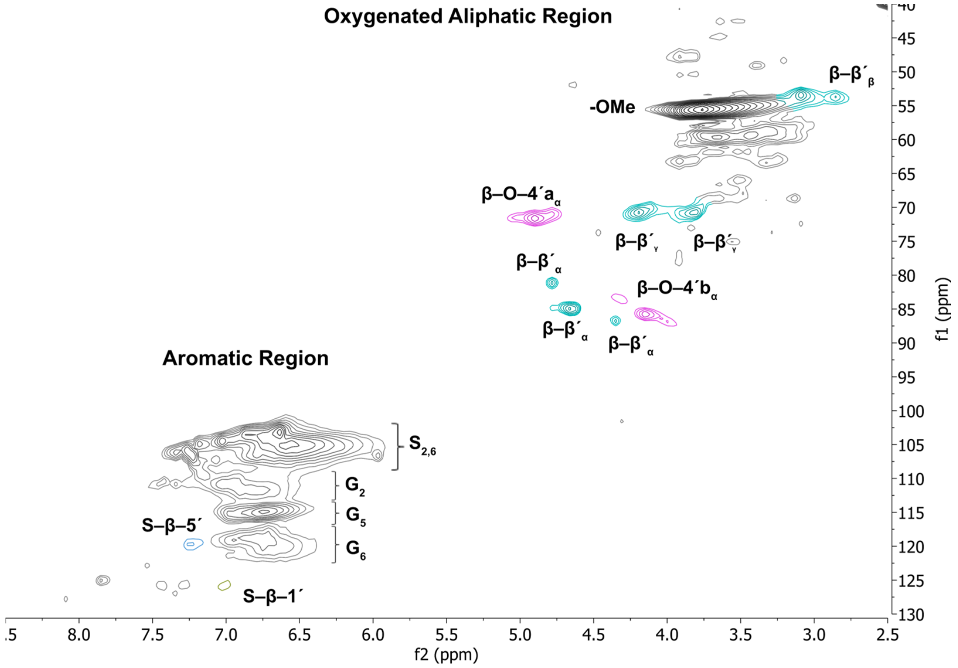

Figure S4. 2D HSQC NMR spectra of reference eucalyptus kraft lignin, with f1 for ^{13}C and f2 for ^{1}H.

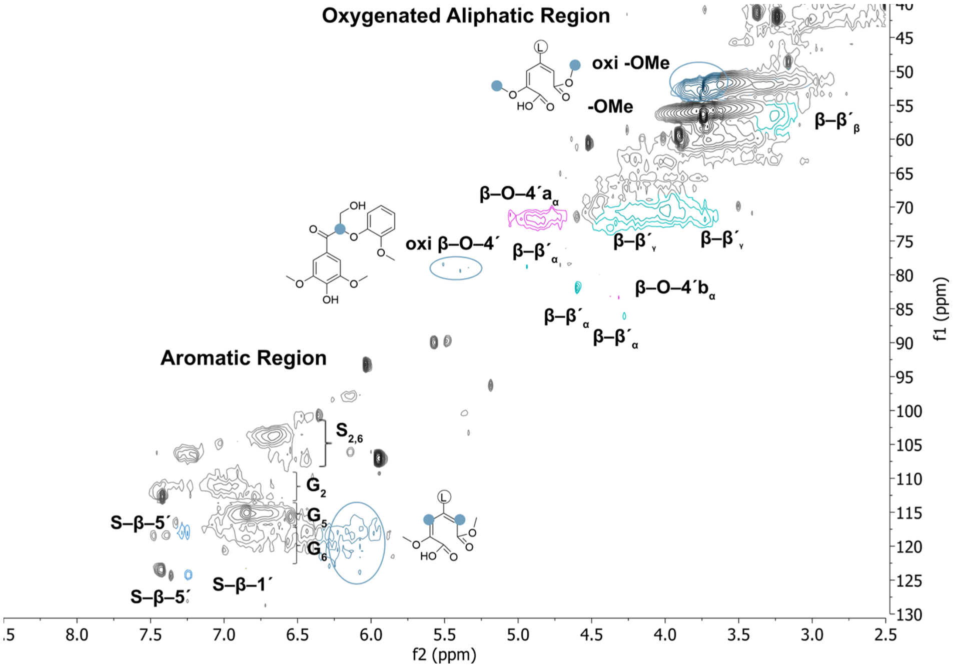

Figure S5. 2D HSQC NMR spectra of oxidized eucalyptus kraft lignin of EtOAc-soluble fraction (OEKL_EtOAc), with f1 for ^{13}C and f2 for ^{1}H.

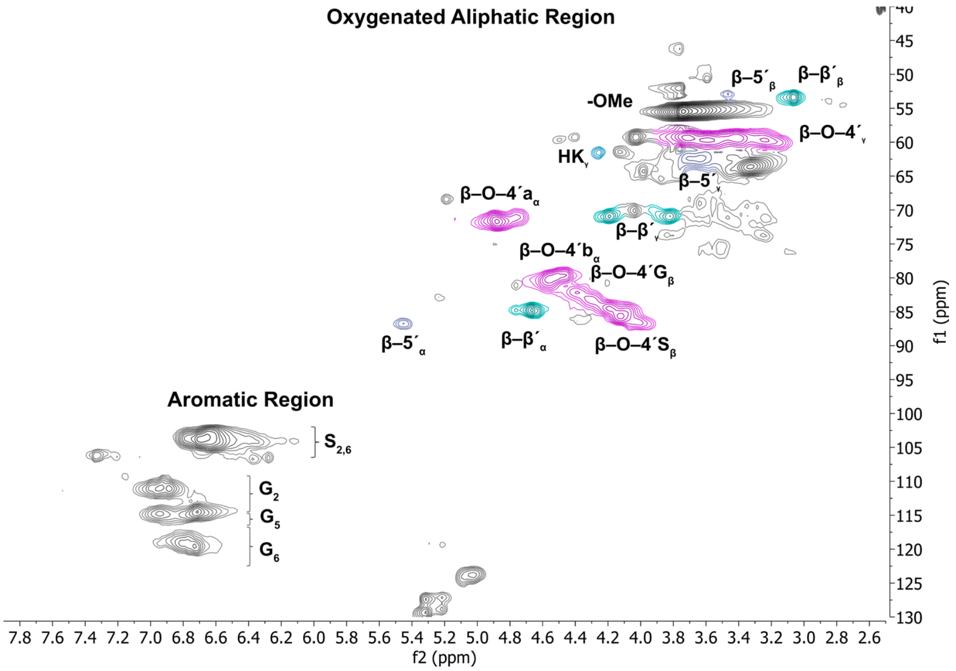

Figure S6. 2D HSQC NMR spectra of reference birch cyclic extracted organosolv lignin, with f1 for ^{13}C and f2 for ^{1}H.

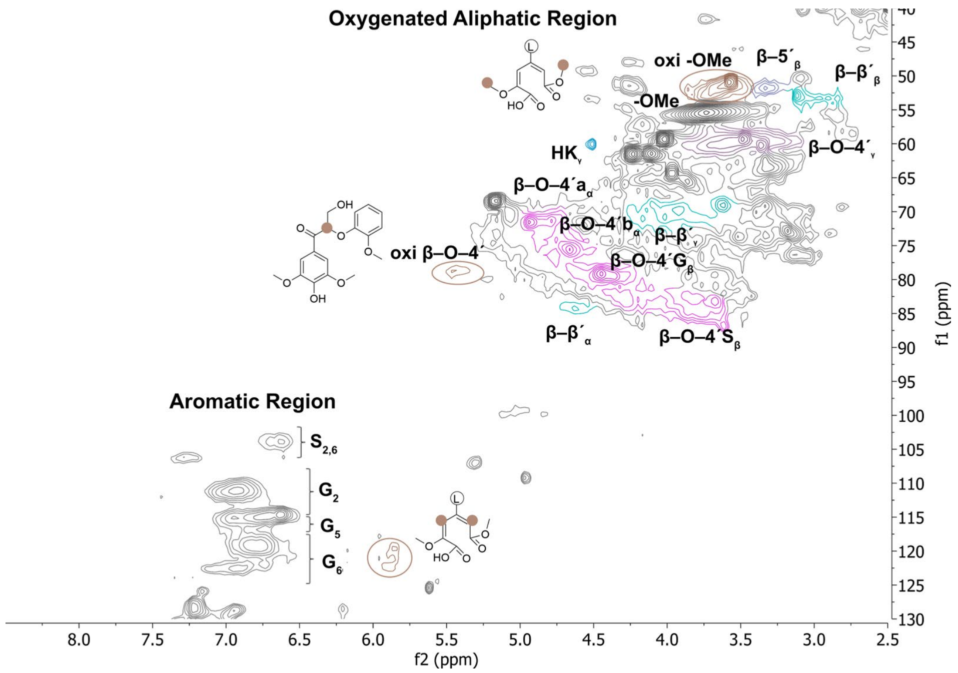

Figure S7. 2D HSQC NMR spectra of oxidized birch cyclic extracted organosolv lignin of precipitated fraction (OBCOL_precip), with f1 for ^{13}C and f2 for ^{1}H.

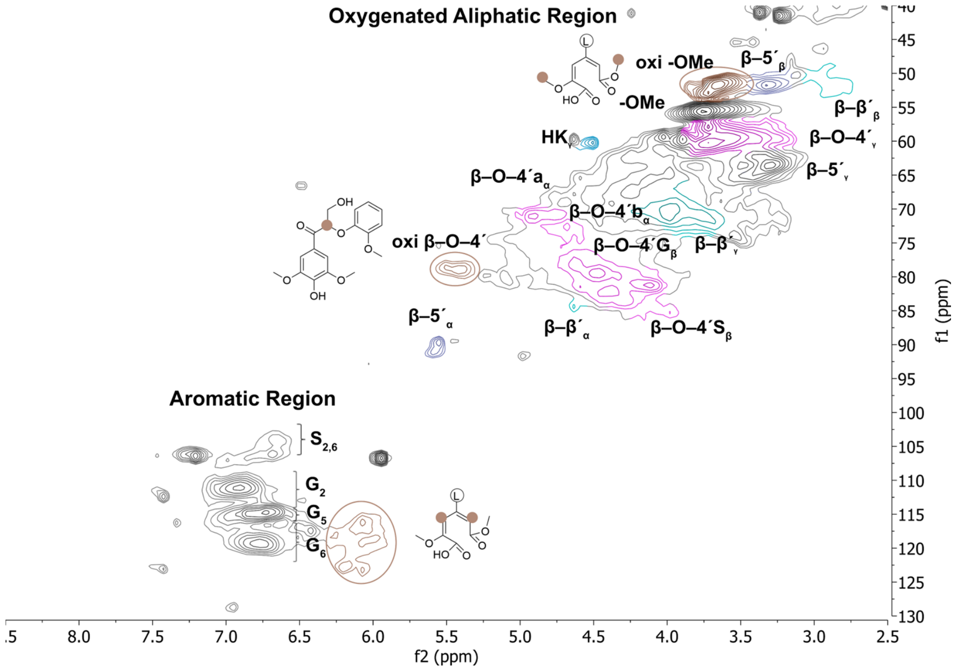

Figure S8. 2D HSQC NMR spectra of oxidized birch cyclic extracted organosolv lignin of EtOAc-soluble fraction (OBCOL_EtOAc), with f1 for ^{13}C and f2 for ^{1}H.

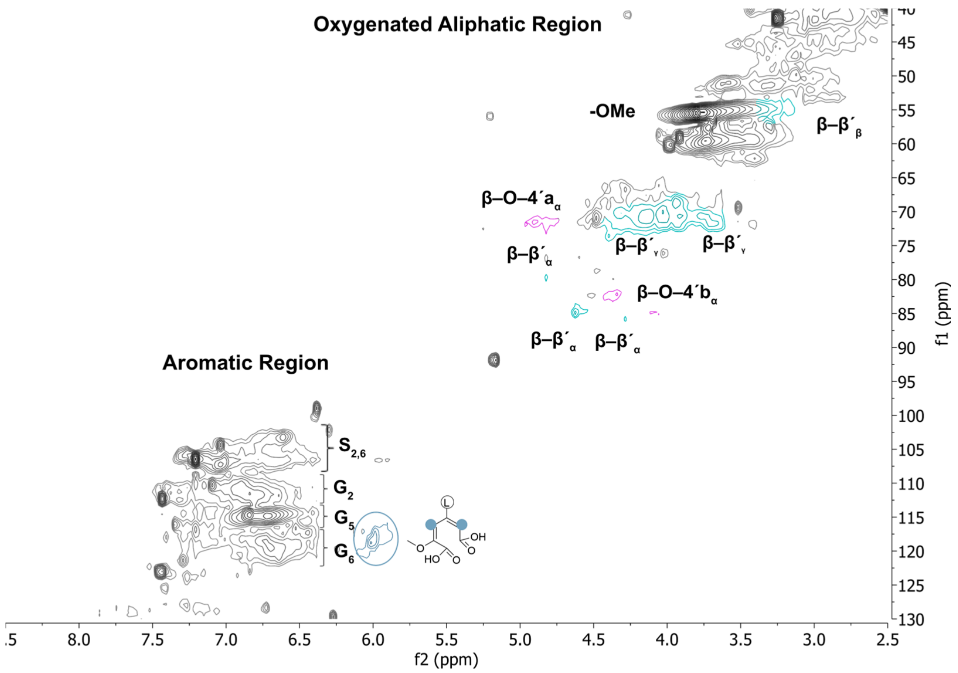

Figure S9. 2D HSQC NMR spectra of oxidized eucalyptus kraft lignin of EtOAc-soluble fraction after alkaline hydrolysis (H_OEKL_EtOAc), with f1 for ^{13}C and f2 for ^{1}H.

2. 2D HMBC NMR Spectra

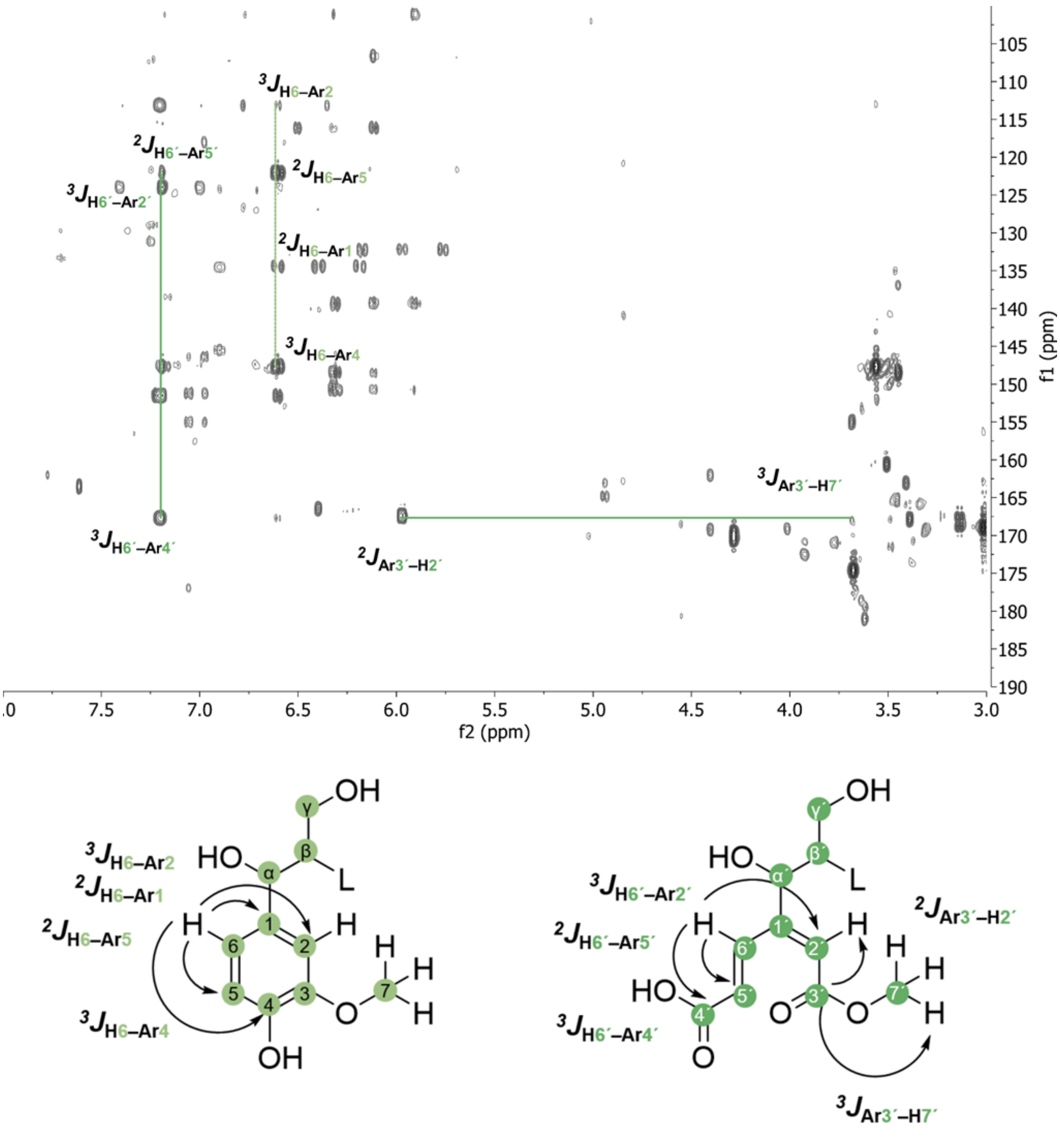

Figure S10. 2D HMBC NMR spectra of oxidized spruce kraft lignin of EtOAc-soluble fraction (OSKL_EtOAc), with f1 for ^{13}C and f2 for ^1H.

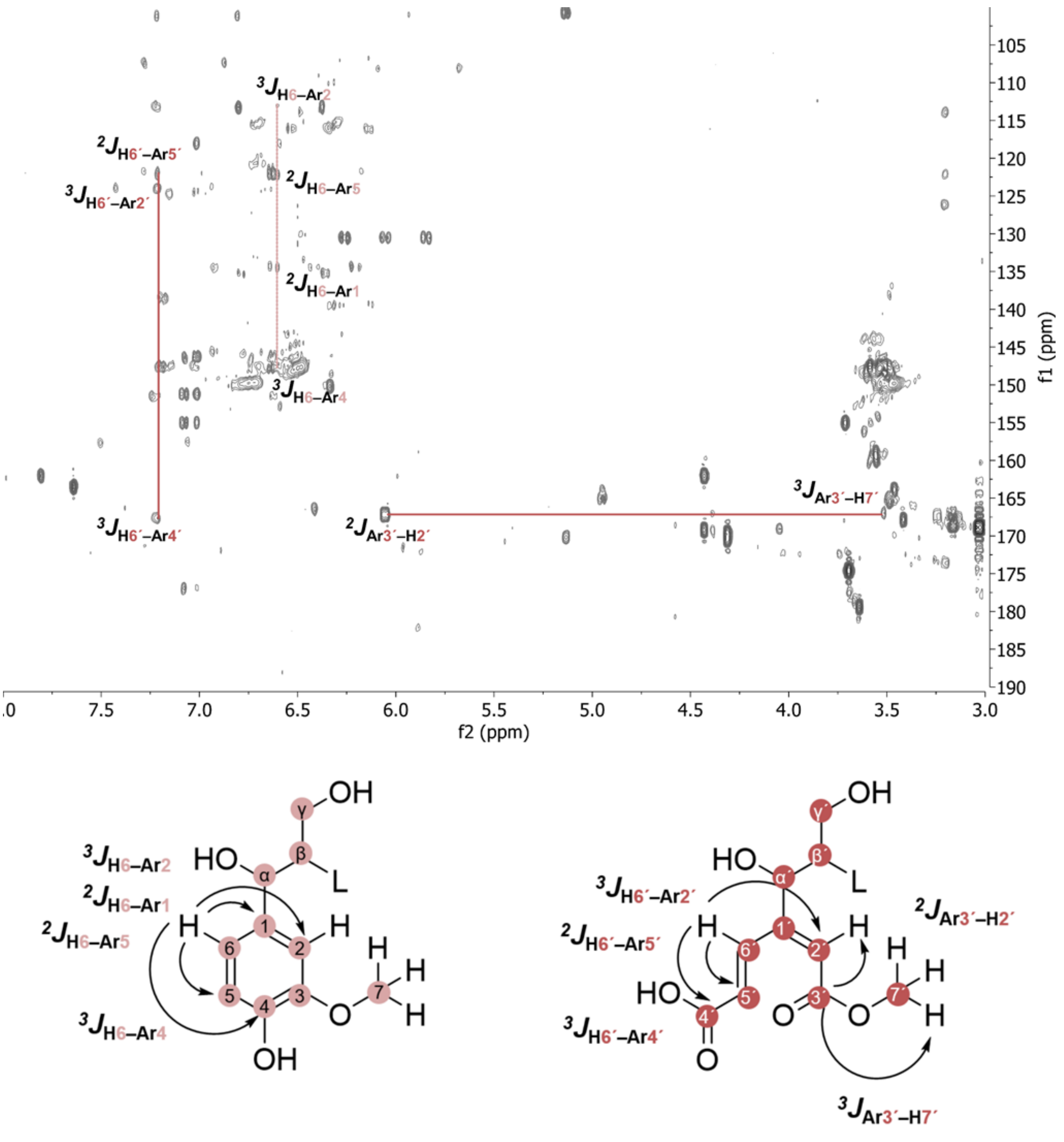

Figure S11. 2D HMBC NMR spectra of oxidized spruce cyclic extracted organosolv lignin of EtOAc-soluble fraction (OSCOL_EtOAc), with f1 for ^{13}C and f2 for ^{1}H.

3. ^{13}C NMR Spectra

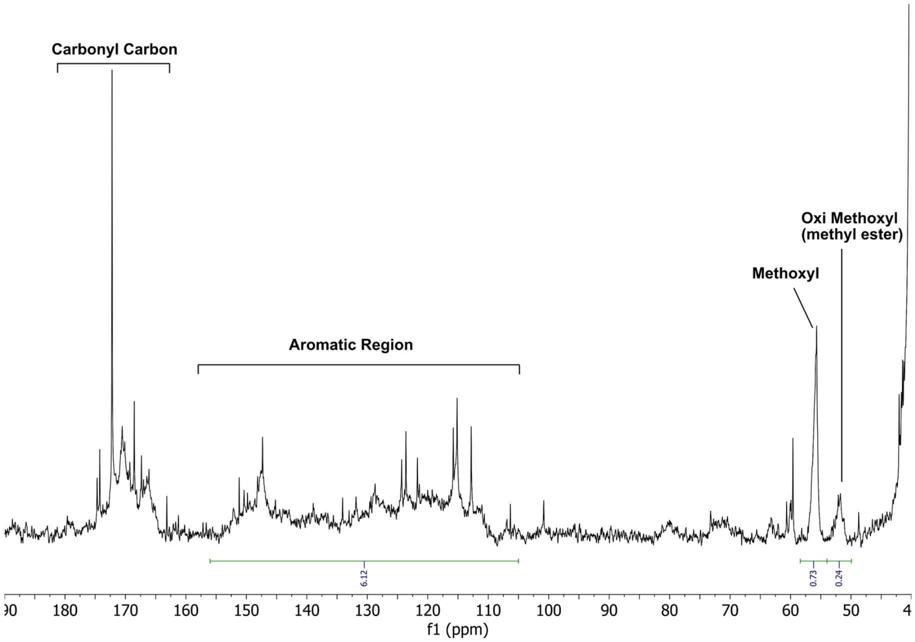

Figure S12. ^{13}C NMR spectra of oxidized spruce kraft lignin of EtOAc-soluble fraction (OSKL_EtOAc).

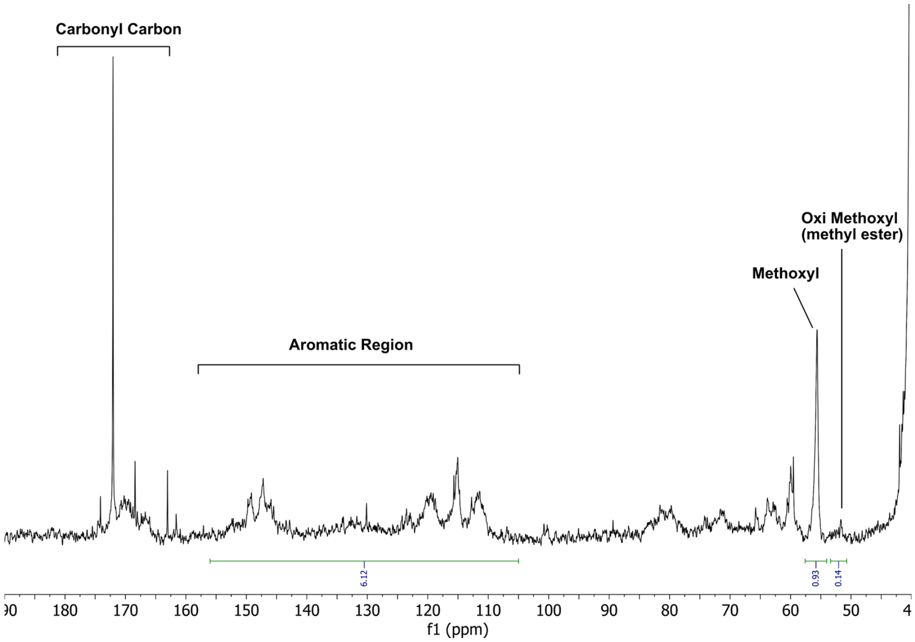

Figure S13. ^{13}C NMR spectra of oxidized spruce cyclic extracted organosolv lignin of EtOAc-soluble fraction (OSCOL_EtOAc).

4. ^{31}P NMR

4.1 Assignment of chemical shifts for quantification of lignin functional groups

Table S1. The chemical shifts of ^{31}P NMR, with water peak from Cl-TMDP at 132.2 ppm as reference.

Functionality	*e*HNDI	Aliphatic –OH	C5-substituted Phenolic –OH	Non-condensed Phenolic –OH		Carboxylic acid –OH
				Guaiacyl –OH	*p*-hydroxy phenyl –OH	
**Chemical shift**	152.1-151.5	149.1-145.1	144.7-141.1	140.6-138.8	138.2-137.3	136.6-133.6

4.2 Functional groups of reference and carboxymethylated lignin samples

Table S2. Quantification of lignin functional groups

Samples	Aliphatic –OH (mmol/g)	C5-substituted Phenolic –OH (mmol/g)	Non-condensed Phenolic –OH (mmol/g)	Carboxylic Acid (mmol/g)
SKL	2.5	1.5	2.4	0.6
OSKL_precip	1.8	0.6	0.7	1.7
OSKL_EtOAc	1.3	0.6	0.9	3.5
H_OSKL_EtOAc	0.8	0.6	1.0	5.2
EKL	1.6	3.5	1.3	0.5
OEKL_EtOAc	1.1	0.9	0.6	3.5
H_OEKL_EtOAc	0.7	0.9	0.9	4.6
SCOL	4.4	0.4	1.2	0.1
OSCOL_precip	3.0	0.5	0.7	0.9
OSCOL_EtOAc	2.9	0.6	1.1	2.7
H_OSCOL_EtOAc	1.5	0.6	1.3	2.9
BCOL	4.0	0.8	0.6	0.2
OBCOL_precip	2.1	0.4	0.5	1.0
OBCOL_EtOAc	1.9	0.7	0.6	3.5
H_OBCOL_EtOAc	1.5	0.8	1.0	4.1

4.3 ^{31}P NMR

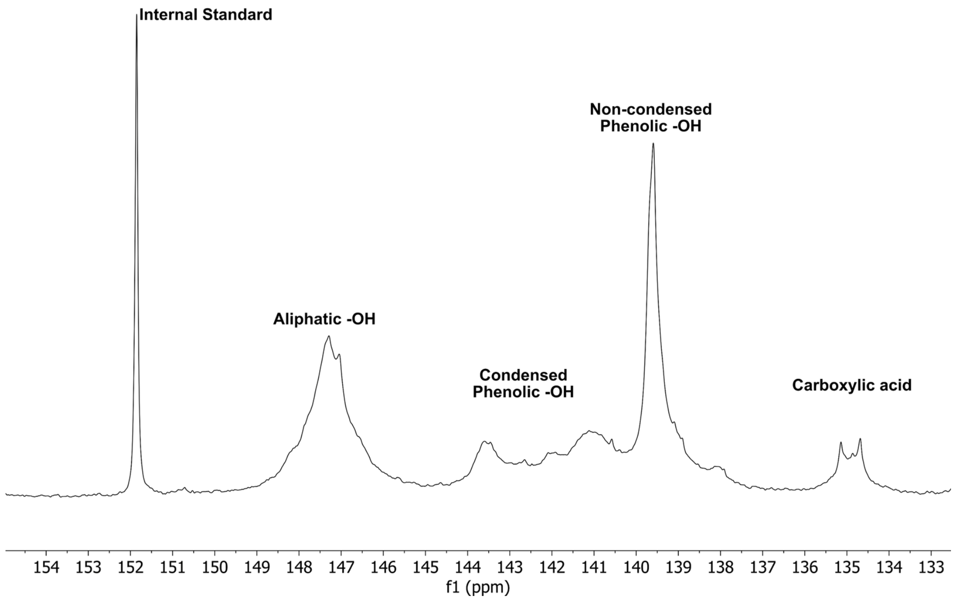

Figure S14. ^{31}P NMR spectra of reference spruce kraft lignin (SKL).

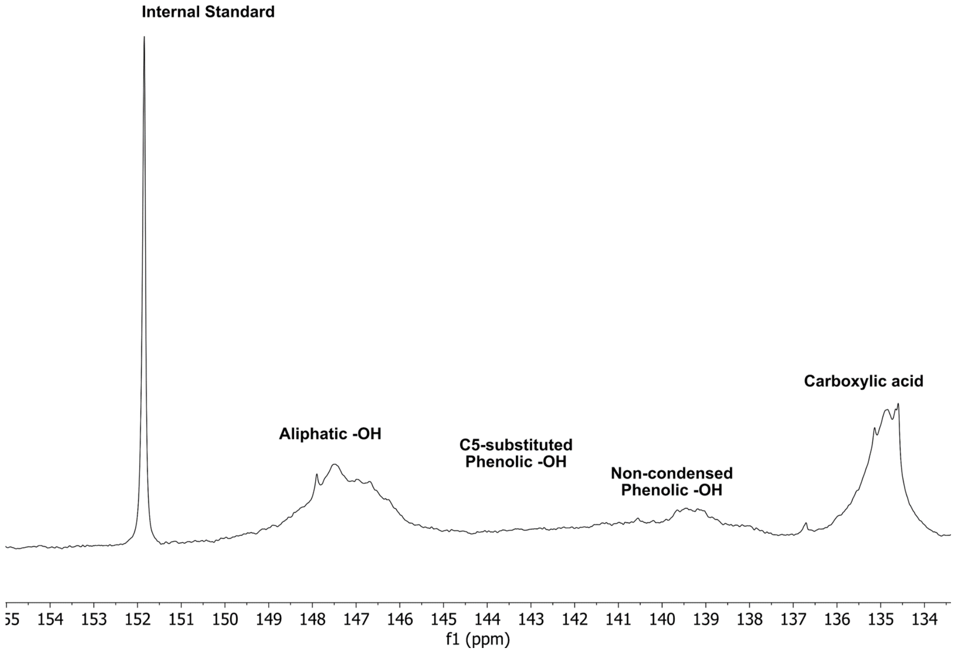

Figure S15. ^{31}P NMR spectra of oxidized spruce kraft lignin of precipitated fraction (OSKL_precip).

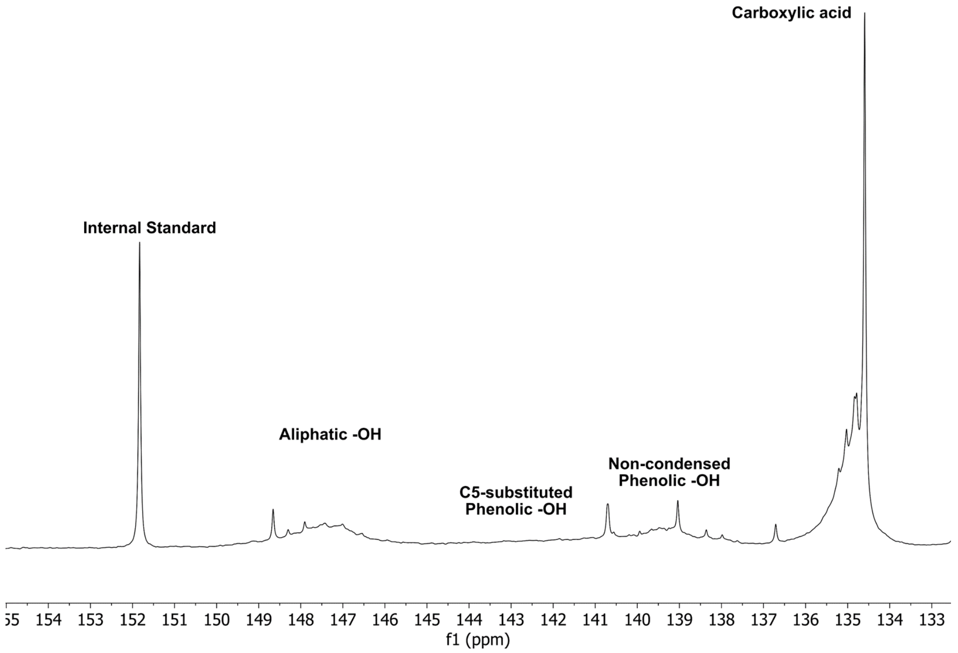

Figure S16. ^{31}P NMR spectra of oxidized spruce kraft lignin of EtOAc-soluble fraction (OSKL_EtOAc).

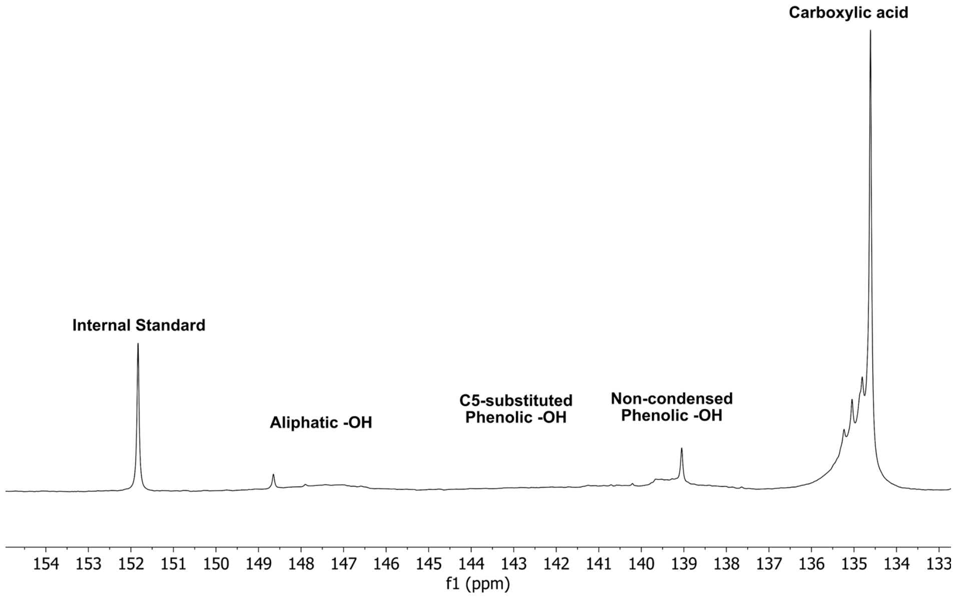

Figure S17. ^{31}P NMR spectra of oxidized spruce kraft lignin of EtOAc-soluble fraction (OSKL_EtOAc) after alkaline hydrolysis.

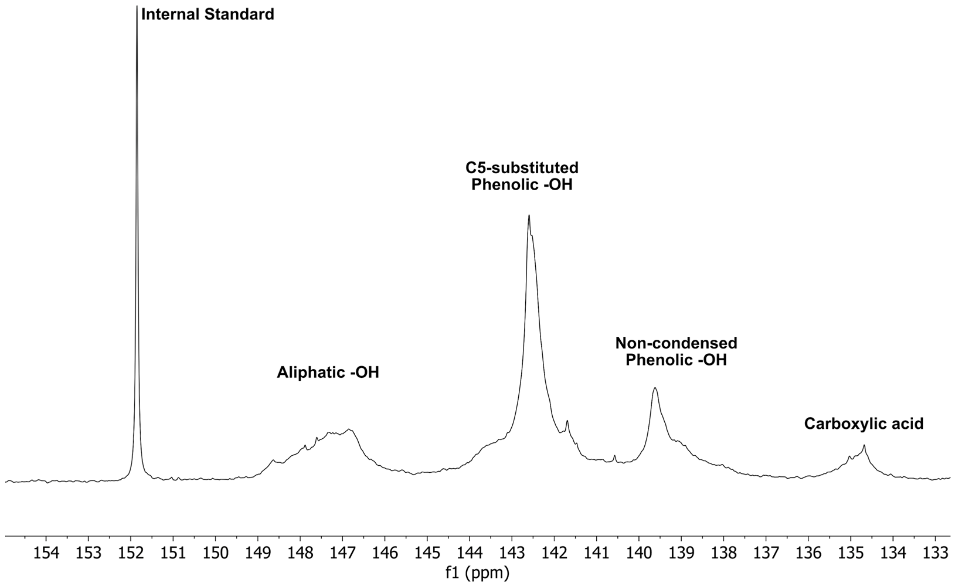

Figure S18. ^{31}P NMR spectra of reference eucalyptus kraft lignin (EKL).

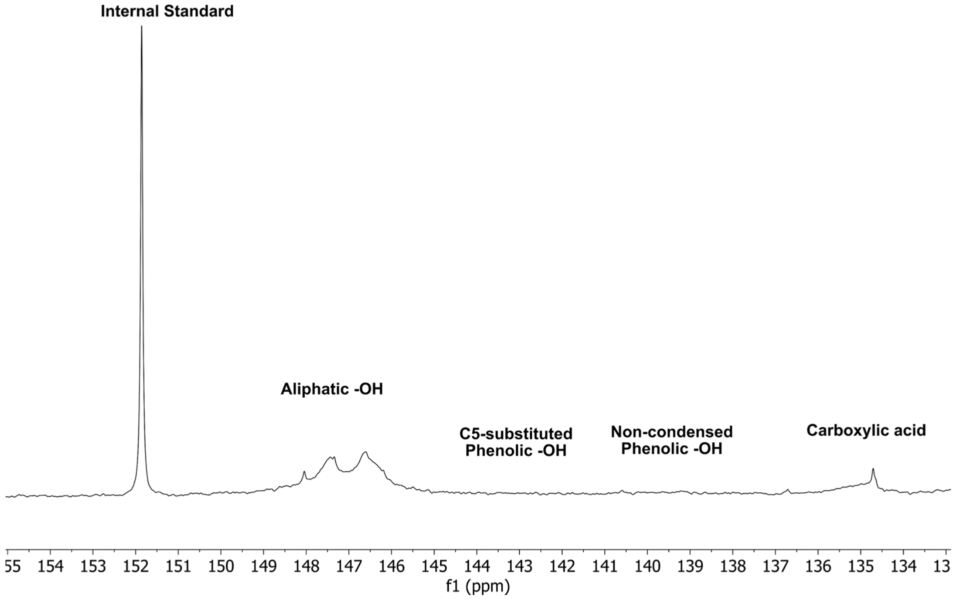

Figure S19. ^{31}P NMR spectra of oxidized eucalyptus kraft lignin of precipitated fraction (OEKL_precip).

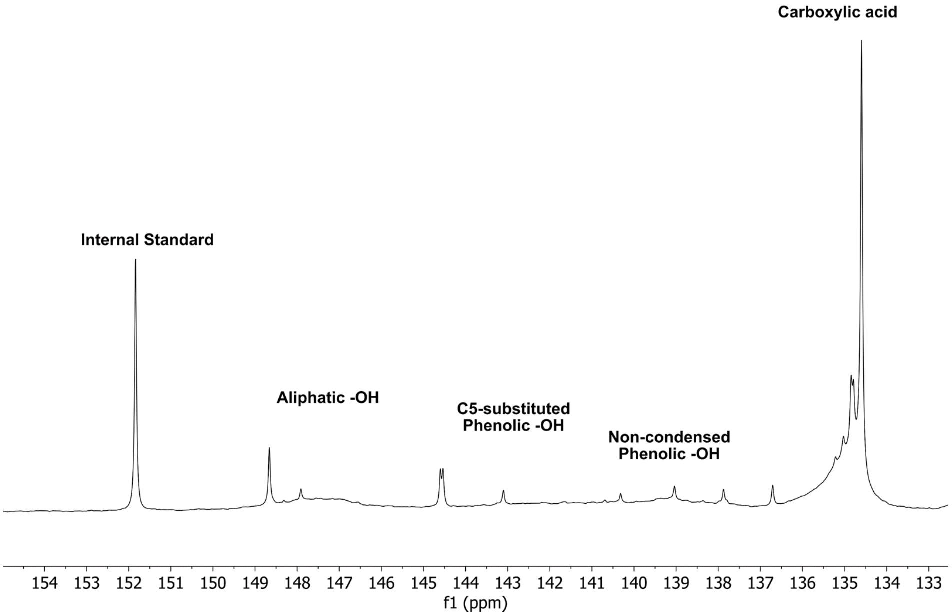

Figure S20. ^{31}P NMR spectra of oxidized eucalyptus kraft lignin of EtOAc-soluble fraction (OEKL_EtOAc).

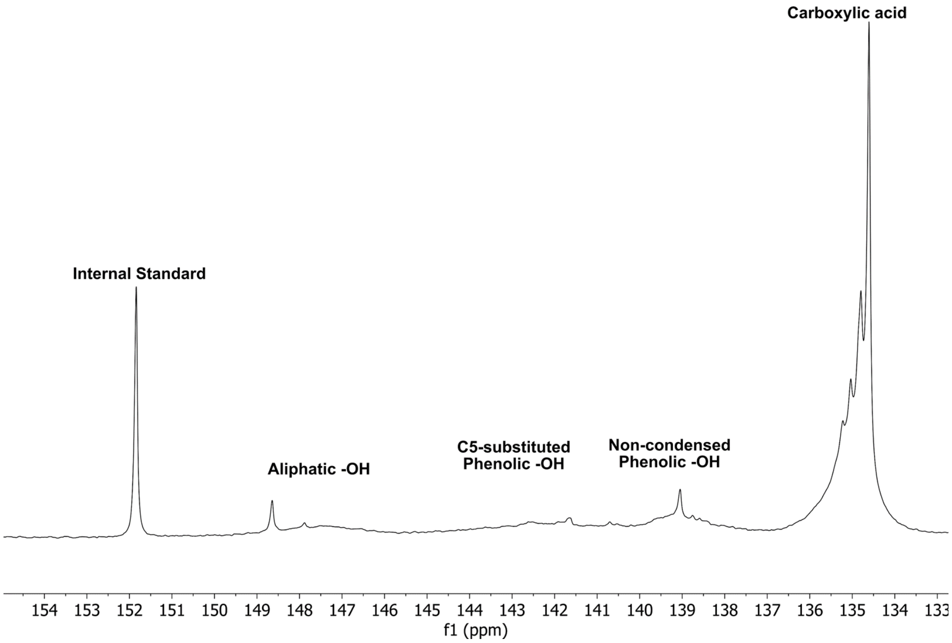

Figure S21. ^{31}P NMR spectra of oxidized eucalyptus kraft lignin of EtOAc-soluble fraction (OEKL_EtOAc) after alkaline hydrolysis.

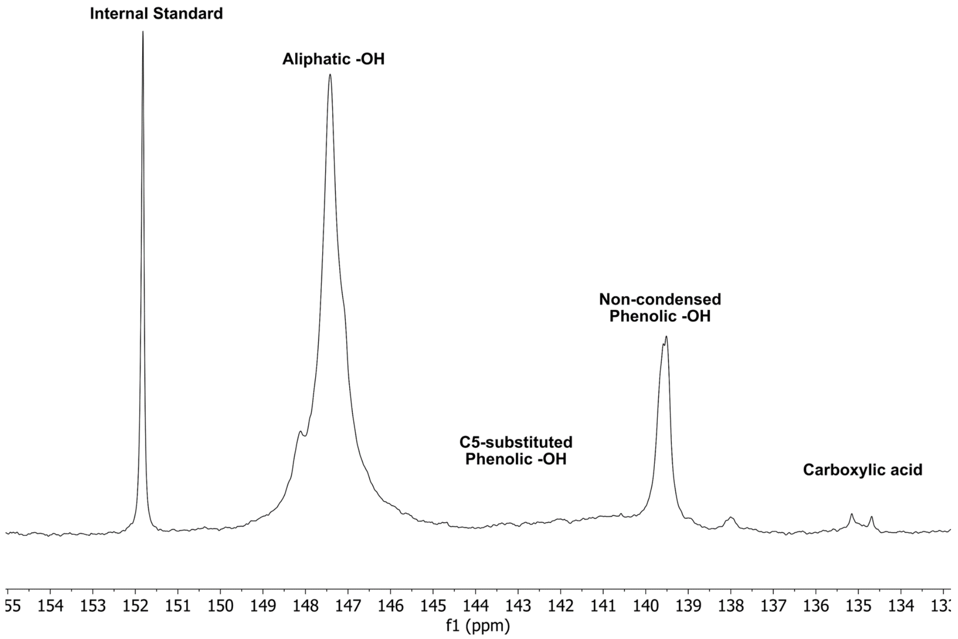

Figure S22. ^{31}P NMR spectra of reference spruce cyclic extracted organosolv lignin (SCOL).

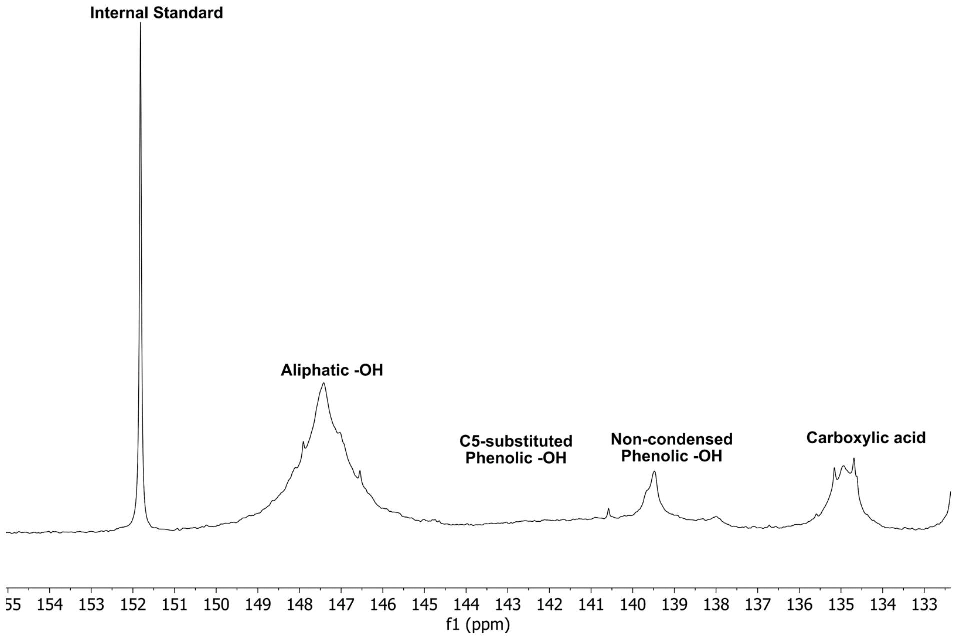

Figure S23. ^{31}P NMR spectra of oxidized spruce cyclic extracted organosolv lignin of precipitated fraction (OSCOL_precip).

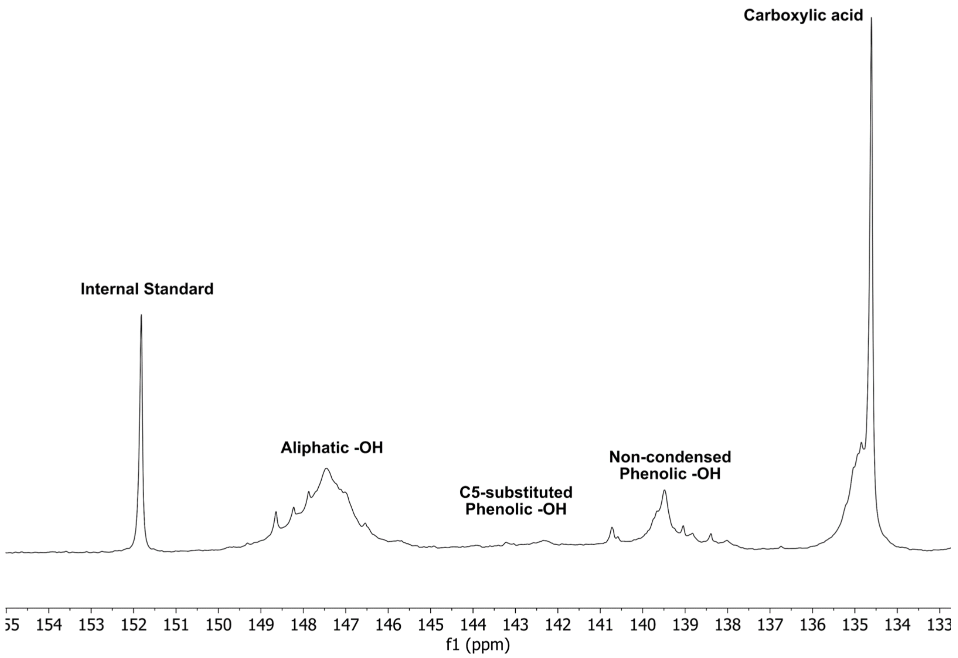

Figure S24. ^{31}P NMR spectra of oxidized spruce cyclic extracted organosolv lignin of EtOAc-soluble fraction (OSCOL_EtOAc).

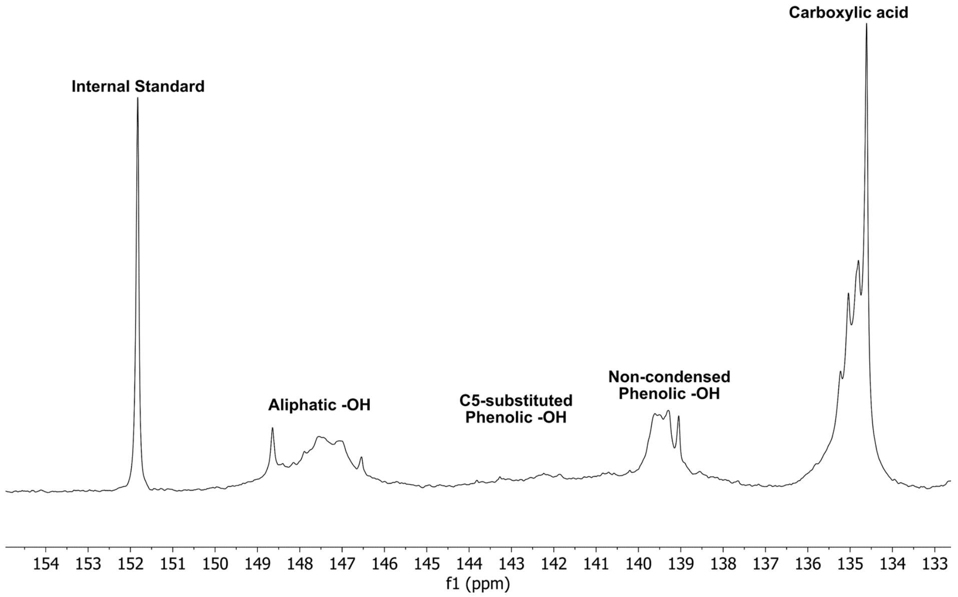

Figure S25. ^{31}P NMR spectra of oxidized spruce cyclic extracted organosolv lignin of EtOAc-soluble fraction (OSCOL_EtOAc) after alkaline hydrolysis.

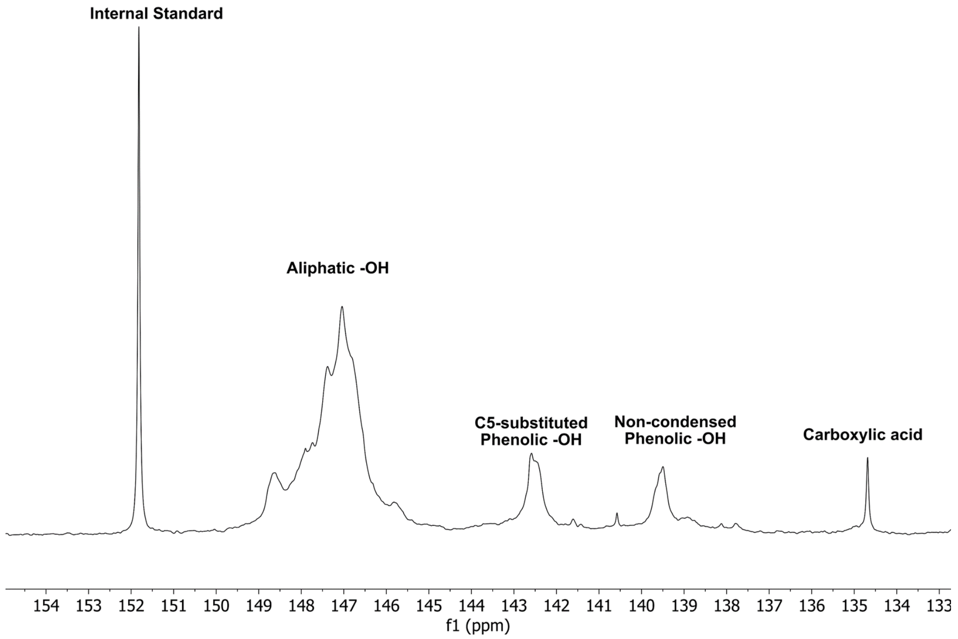

Figure S26. ^{31}P NMR spectra of reference birch cyclic extracted organosolv lignin (BCOL).

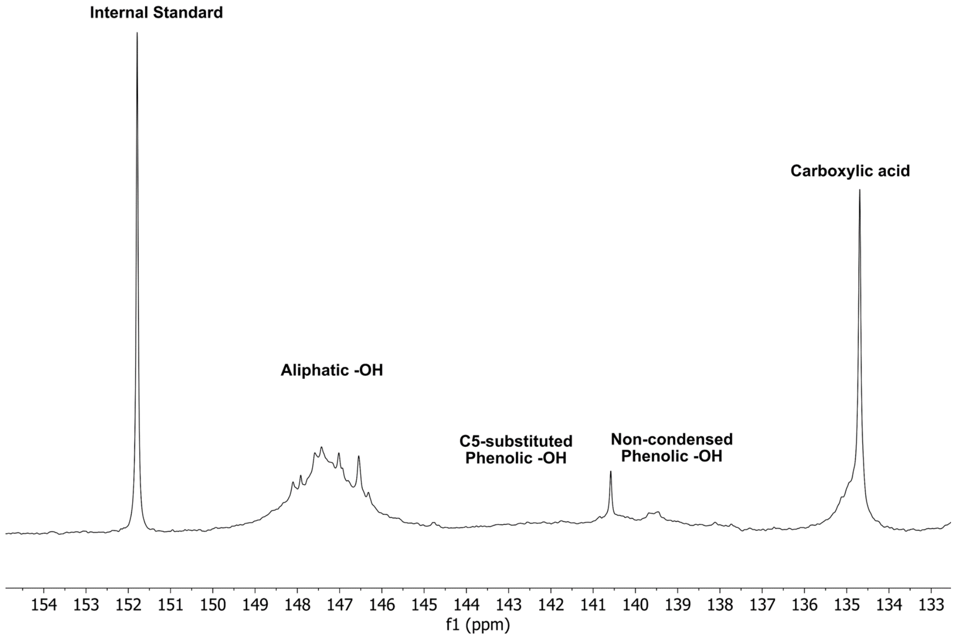

Figure S27. ^{31}P NMR spectra of oxidized birch cyclic extracted organosolv lignin of precipitated fraction (OBCOL_precip).

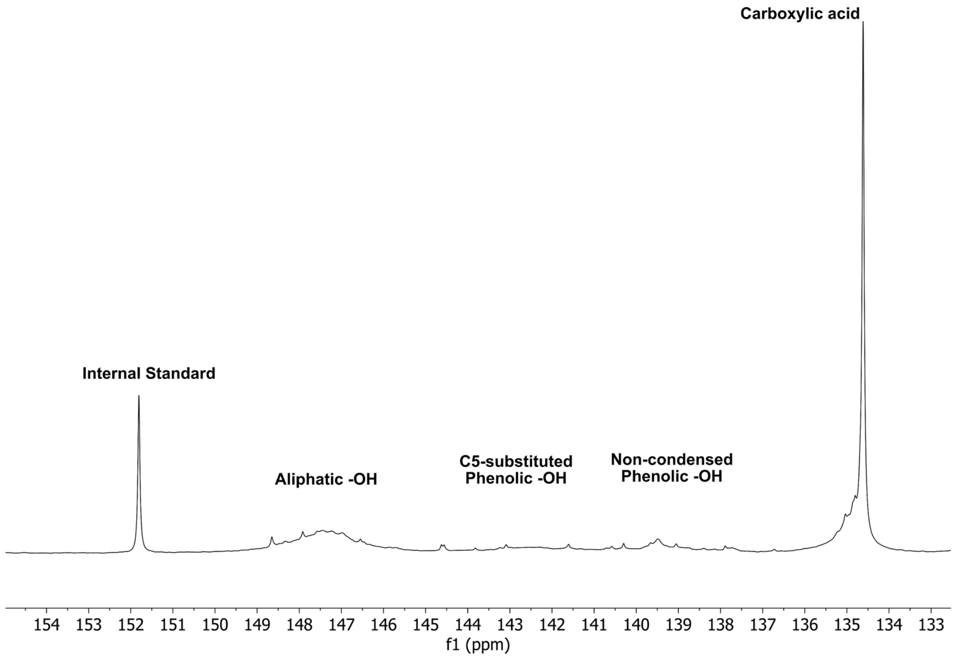

Figure S28. ^{31}P NMR spectra of oxidized birch cyclic extracted organosolv lignin of EtOAc-soluble fraction (OBCOL_EtOAc).

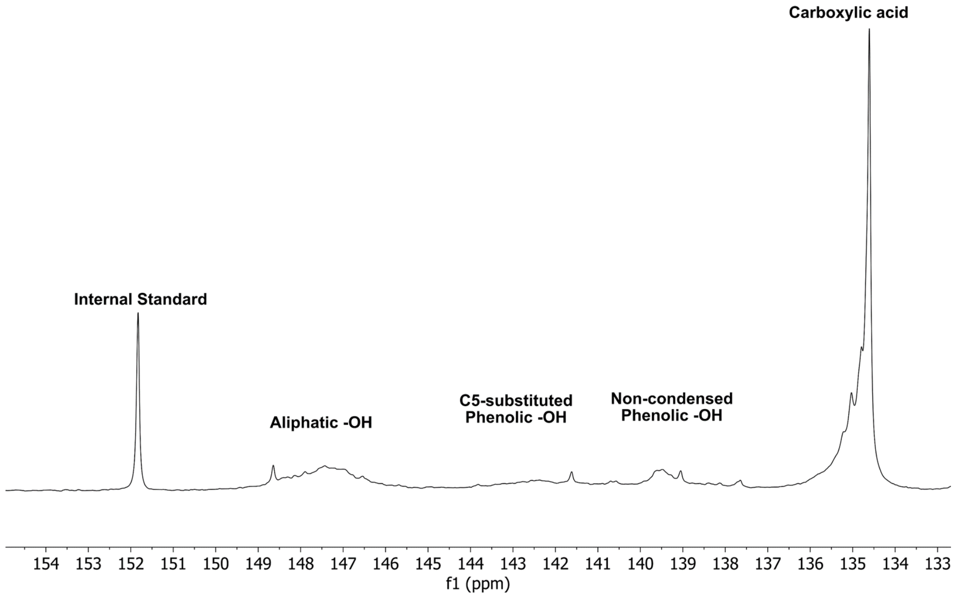

Figure S29. ^{31}P NMR spectra of oxidized birch cyclic extracted organosolv lignin of EtOAc-soluble fraction (OBCOL_EtOAc) after alkaline hydrolysis.

5. SEC

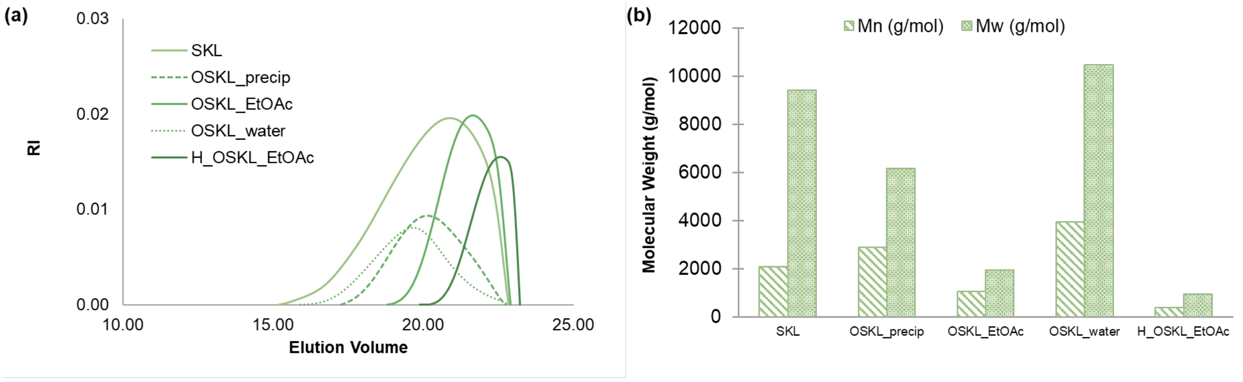

Figure S30. (a) Elution volume; (b) molecular weight of SKL, OSKL_precip, OSKL_EtOAc and H_OSKL_EtOAc.

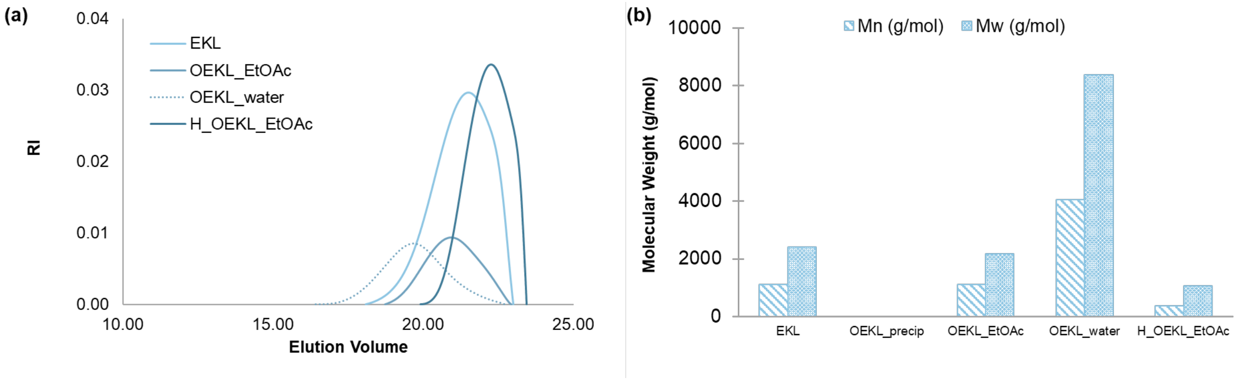

Figure S31. (a) Elution volume; (b) molecular weight of EKL, OEKL_precip, OEKL_EtOAc and H_OEKL_EtOAc.

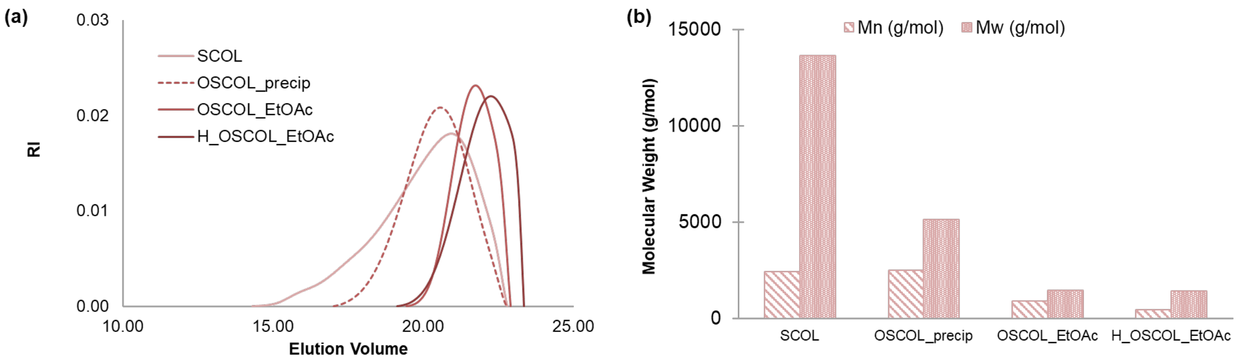

Figure S32. (a) Elution volume; (b) molecular weight of SCOL, OSCOL_precip, OSCOL_EtOAc and H_OSCOL_EtOAc.

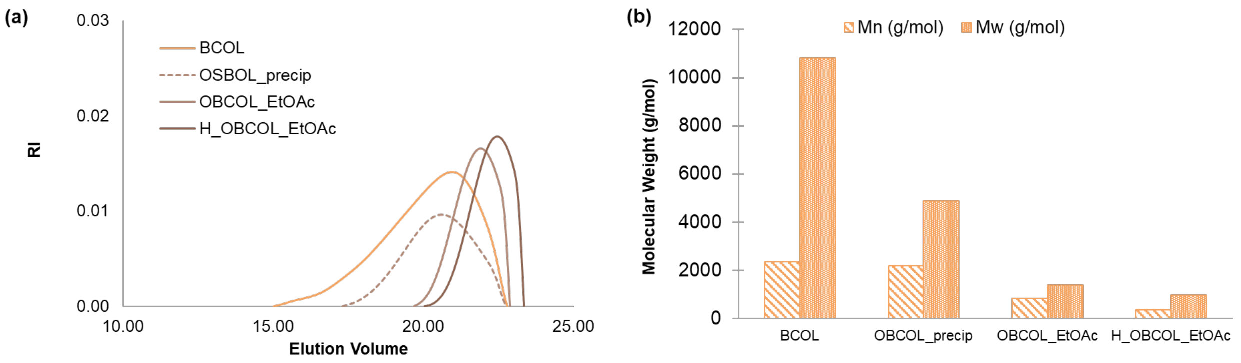

Figure S33. (a) Elution volume; (b) molecular weight of BCOL, OBCOL_precip, OBCOL_EtOAc and H_OBCOL_EtOAc.

6. Yield of Oxidized Lignin after Alkaline Hydrolysis Treatment

Table S3. Yield of both precipitated and EtOAc-soluble fraction of oxidized lignin samples after alkaline hydrolysis treatment.

Sample	Precipitated Fraction		EtOAc-soluble Fraction	
	Abbreviation	Yield (%)[a]	Abbreviation	Yield (%)[b]
OSKL_EtOAc	H_OSKL_precip	30%	H_OSKL_EtOAc	56%
OEKL_EtOAc	H_OEKL_precip	22%	H_OEKL_EtOAc	51%
OSCOL_EtOAc	H_OSCOL_precip	22%	H_OSCOL_EtOAc	62%
OBCOL_EtOAc	H_OBCOL_precip	7.5%	H_OBCOL_EtOAc	60%

[a] The yield of precipitated fraction was calculated based on the dry mass of precipitated fraction after workup step of alkaline hydrolysis treatment, compared to initial mass of EtOAc-soluble fraction of oxidized lignin before hydrolysis.

[b] The yield of EtOAc-soluble fraction was calculated based on the dry mass of EtOAc-soluble fraction after EtOAc extraction of water phase from alkaline hydrolysis treatment, compared to initial mass of EtOAc-soluble fraction of oxidized lignin before hydrolysis.

7. Analysis of Water-Soluble Fraction of Oxidized Lignin

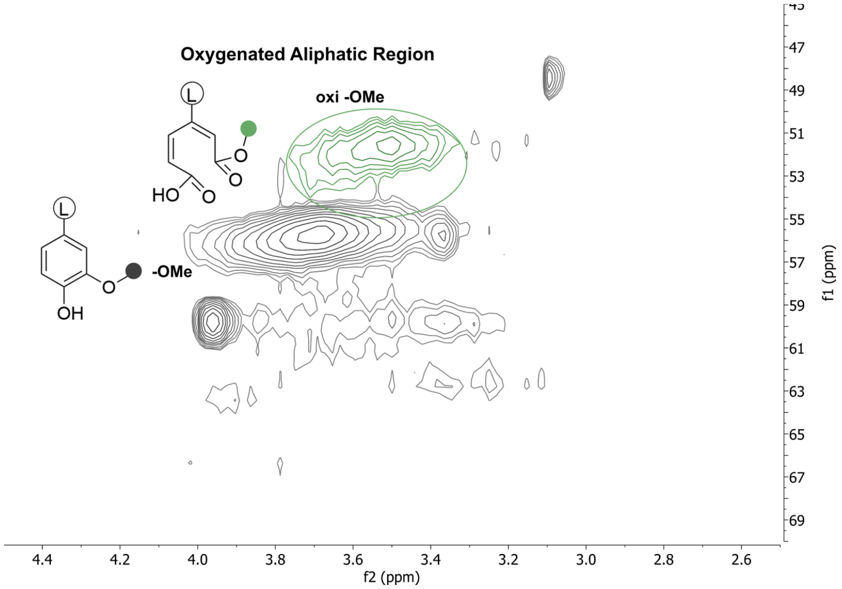

Figure S34. 2D HSQC NMR spectra of oxidized spruce kraft lignin of Ca^{2+} ion-extracted water-soluble, with f1 for ^{13}C and f2 for ^{1}H.

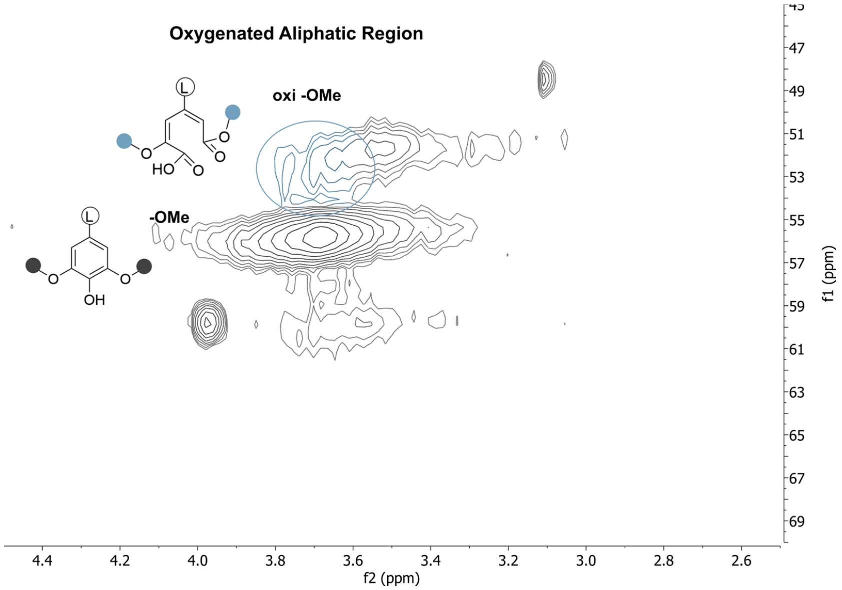

Figure S35. 2D HSQC NMR spectra of oxidized eucalyptus kraft lignin of Ca^{2+} ion-extracted water-soluble, with f1 for ^{13}C and f2 for ^{1}H.
